# Innate programmable DNA binding by CRISPR-Cas12m effectors enable efficient base editing

**DOI:** 10.1093/nar/gkae016

**Published:** 2024-01-23

**Authors:** Greta Bigelyte, Brigita Duchovska, Rimante Zedaveinyte, Giedrius Sasnauskas, Tomas Sinkunas, Indre Dalgediene, Giedre Tamulaitiene, Arunas Silanskas, Darius Kazlauskas, Lukas Valančauskas, Julene Madariaga-Marcos, Ralf Seidel, Virginijus Siksnys, Tautvydas Karvelis

**Affiliations:** Institute of Biotechnology, Life Sciences Center, Vilnius University, Vilnius LT-10257, Lithuania; Institute of Biotechnology, Life Sciences Center, Vilnius University, Vilnius LT-10257, Lithuania; Institute of Biotechnology, Life Sciences Center, Vilnius University, Vilnius LT-10257, Lithuania; Institute of Biotechnology, Life Sciences Center, Vilnius University, Vilnius LT-10257, Lithuania; Institute of Biotechnology, Life Sciences Center, Vilnius University, Vilnius LT-10257, Lithuania; Institute of Biotechnology, Life Sciences Center, Vilnius University, Vilnius LT-10257, Lithuania; Institute of Biotechnology, Life Sciences Center, Vilnius University, Vilnius LT-10257, Lithuania; Institute of Biotechnology, Life Sciences Center, Vilnius University, Vilnius LT-10257, Lithuania; Institute of Biotechnology, Life Sciences Center, Vilnius University, Vilnius LT-10257, Lithuania; Institute of Biotechnology, Life Sciences Center, Vilnius University, Vilnius LT-10257, Lithuania; Peter Debye Institute for Soft Matter Physics, University of Leipzig, Leipzig 04103, Germany; Peter Debye Institute for Soft Matter Physics, University of Leipzig, Leipzig 04103, Germany; Institute of Biotechnology, Life Sciences Center, Vilnius University, Vilnius LT-10257, Lithuania; Institute of Biotechnology, Life Sciences Center, Vilnius University, Vilnius LT-10257, Lithuania

## Abstract

Cas9 and Cas12 nucleases of class 2 CRISPR-Cas systems provide immunity in prokaryotes through RNA-guided cleavage of foreign DNA. Here we characterize a set of compact CRISPR-Cas12m (subtype V-M) effector proteins and show that they provide protection against bacteriophages and plasmids through the targeted DNA binding rather than DNA cleavage. Biochemical assays suggest that Cas12m effectors can act as roadblocks inhibiting DNA transcription and/or replication, thereby triggering interference against invaders. Cryo-EM structure of *Gordonia otitidis* (Go) Cas12m ternary complex provided here reveals the structural mechanism of DNA binding ensuring interference. Harnessing GoCas12m innate ability to bind DNA target we fused it with adenine deaminase TadA-8e and showed an efficient A-to-G editing in *Escherichia coli* and human cells. Overall, this study expands our understanding of the functionally diverse Cas12 protein family, revealing DNA-binding dependent interference mechanism of Cas12m effectors that could be harnessed for engineering of compact base-editing tools.

## Graphical Abstract

**Graphical Abstract:**
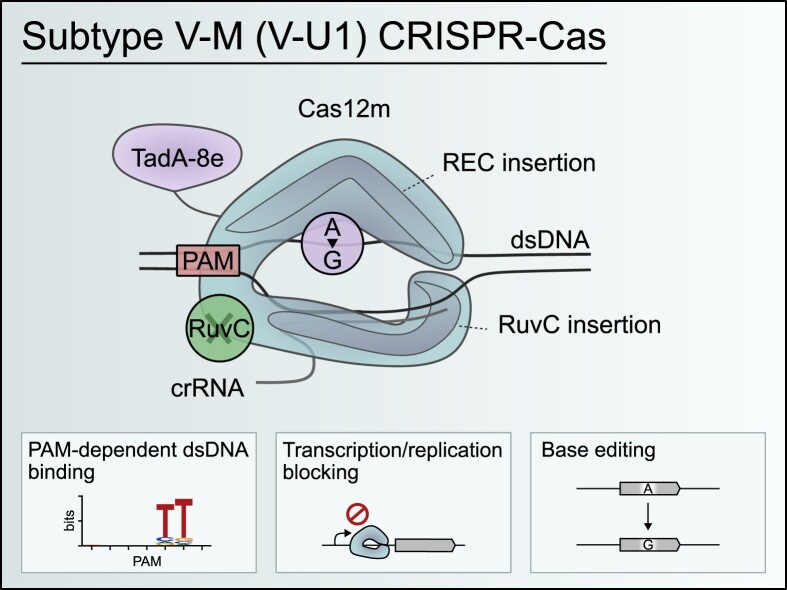

## Introduction

CRISPR (clustered regularly interspaced short palindromic repeats)-Cas (CRISPR-associated) systems protect prokaryotes from mobile genetic elements (MGEs) (1). Interference against invading MGEs is provided by Cas effector complexes that use CRISPR RNAs (crRNAs) to target foreign DNA or RNA in a sequence-specific manner. Based on the composition of the effector complex, CRISPR-Cas systems are classified into 2 classes. In class 1 CRISPR-Cas (types I, III, and IV), effector complexes are composed of multiple Cas proteins, whereas class 2 systems (type II, V, and VI) encompass a single Cas effector (2,3). DNA targeting class 2 effector complexes, exemplified by the Cas9 (type II) and Cas12 (type V) proteins, are of particular interest, due to their prevalent use in genome editing field (4,5).

Extensive bioinformatic and experimental mining of Cas9 and Cas12 orthologs have led to the discovery of novel protein variants and CRISPR-Cas systems (2,3,6–9). While the Cas9 family exhibits limited architectural and functional diversity, with the majority of its variants being 1000–1500 aa in size and sharing HNH and RuvC domains (6,7), the Cas12 family, containing a single RuvC domain, is highly diverse in both function and size (400–1500 aa) (8,10–14). The smallest Cas12 effectors (400–600 aa), initially classified as subtypes V-U1 to V-U5 (3), are of particular interest due to their potential to be delivered into the cells using limited capacity adeno-associated viruses (AAVs) which are the preferred delivery vectors for *in vivo* applications. Typical subtype V-U1 to V-U5 CRISPR-Cas systems lack genes involved in spacer acquisition and encode Cas12 effectors with high sequence similarity to TnpB proteins (3). It has been suggested that these Cas12 effectors are evolutionary intermediates between TnpB nucleases found in IS*200*/IS*605* bacterial insertion sequences and the more complex Cas12 family effectors (2,3,15,16).

Based on the sequence similarity, the Cas12 proteins of V-U2, V-U3 and V-U4 subtypes have recently been assigned to the V-F subtype (2). Cas12f effectors are PAM-dependent RNA-guided DNA nucleases that require a complex guide RNA (gRNA) and function as dimers (11,17–19). Despite the efficient DNA cleavage *in vitro*, they typically show low activity in eukaryotic cells and therefore require additional protein and/or gRNA engineering for genome editing applications (17,20–24). Catalytically-dead Cas12 effectors from the subtype V-U5 systems (assigned to subtype V-K) form multiprotein effector complexes with Tn7-like transposase and are involved in RNA-guided transposon ‘homing’ (25–27).

To date, the only V-U1 Cas12 effector containing an inactivated RuvC domain (denoted to subtype V-M, and named Cas12m) from *Mycolicibacterium mucogenicum* (Mm) has been characterized (28,29). It has a relatively small size (596 aa) and provides DNA silencing through the RNA-guided target DNA binding (28,29). Here, we biochemically characterize a set of Cas12m effectors and show that they display RNA-guided PAM-dependent DNA binding activity and exhibit different PAM preferences. We also report the cryo-EM structure of *Gordonia otitidis* (Go) Cas12m-crRNA-DNA ternary complex that reveals the structural mechanism of Cas12m binding. Finally, by fusing GoCas12m with adenine deaminase, we demonstrate the possibility to engineer an efficient base editor that shows a narrow PAM-proximal editing window.

## Materials and methods

### Identification of CRISPR-Cas V-U1 family members

The initial search of CRISPR-Cas type V-U1 candidates, recently assigned to type V-M (29), was conducted in the NCBI nr (30), IMG/M (31), and IMG/VR (32) databases using online versions of BLASTP (E-value <1e-5) (33) and a previously identified type V-U1 family effector protein from *Gordonia otitidis* NBRC 100426 (GAB36148.1) (2,3) as a query. Next, from the retrieved candidates, we selected the Cas12m variant from *Thermanaerosceptrum fracticalcis* DRI-13 (QNB45423.1) and performed a second round of search using the same strategy. The retrieved proteins from both searches were combined and variants larger than 300 amino acids (aa) were retained for further analysis. The obtained Cas12m candidates were then clustered using online version of MMseqs2 (0.95 identity, 0.8 coverage) (34), and only the largest variant was retained from each cluster, resulting in a non-redundant list of 134 Cas12m protein variants (Supplementary Table S1). Nucleotide sequences up to 2.5 kb upstream and downstream of the Cas12m encoding genes were analyzed using an online version of the CRISPRCasFinder tool (35) to detect CRISPR region. Finally, 8 CRISPR-Cas type V-M systems were selected for further analysis (Supplementary Table S2).

### Cloning of Cas12m expression vectors

The genes of selected Cas12m proteins were synthesized and cloned into pBAD expression vectors (Twist Biosciences) fused with N-terminal Twin-Strep-10×His-MBP purification tag encoding sequences (pTK200-203, pTK210-211, TK216-217). Cas12m expression vectors containing a minimal CRISPR region, consisting of two repeats and a single spacer between them, were cloned using Golden Gate strategy (pTK205-208, pTK213-214, pTK218-219). Specifically, the fragments of the minimal CRISPR region with the native adjacent sequences (up to 300 bp downstream the last repeat) were ordered as gBlocks (IDT). A plasmid encoding Cas12m and fragments of its respective CRISPR region was incubated with FD BpiI (Thermo Fisher Scientific) and T4 DNA ligase (Thermo Fisher Scientific) in 1× FastDigest buffer supplemented with 0.5 mM ATP (Thermo Fisher Scientific) for 30 cycles at 37°C/5 min and 16°C/5 min, followed by 65°C 10 min. The plasmids were propagated in *E. coli* DH5α and purified using GeneJET Plasmid Miniprep Kit (Thermo Fisher Scientific). Descriptions and links to protein and plasmid sequences can be found in Supplementary Table S3 and S4.

### Expression and purification of Cas12 effectors and their RNP complexes

For Cas12m and Cas12a protein purification, *E. coli* DH10B strain was transformed with expression vectors (pMBP-AsCas12a (gift from Jennifer Doudna, Addgene plasmid #113430), pGB060 (produced from pMBP-AsCas12a by introducing D908A modification, using Phusion Site-Directed Mutagenesis Kit (Thermo Fisher Scientific), pTK200, pTK203; Supplementary Table S4). The cells were grown in LB broth supplemented with ampicillin (100 μg/ml) at 37°C until OD_600_ reached 0.8. The protein expression was induced with 0.2% L-arabinose and the cells were grown for additional 16 h at 16°C. Cells were pelleted by centrifugation, resuspended in 20 mM Tris–HCl (pH 8.0 at 25°C), 1 M NaCl, 5 mM 2-mercaptoethanol, 25 mM imidazole and 5% (v/v) glycerol buffer, and disrupted by sonication. Cell debris was removed by centrifugation and the remaining supernatant was loaded on Ni^2+^-charged HiTrap chelating HP column (GE Healthcare). The proteins of interest were eluted by increasing imidazole concentration from 25 mM to 500 mM in 20 mM Tris–HCl (pH 8.0 at 25°C), 500 mM NaCl, 5 mM 2-mercaptoethanol and 5% (v/v) glycerol buffer. The fractions containing the protein of interest were pooled and the 10×His-MBP tag was cleaved by incubating with TEV protease overnight at 4°C. To remove the cleaved 10×His-MBP tag and TEV protease, reaction mixtures were loaded onto a HiTrap heparin HP 5 column (GE Healthcare) and a linear gradient of increasing NaCl concentration (250–1000 mM) was used for elution. The collected fractions with Cas12 proteins were dialyzed against 20 mM Tris–HCl (pH 8.0 at 25°C), 0.5 M NaCl, 2 mM DTT and 50% (v/v) glycerol buffer, and stored at −20°C. For Cas12m RNP complex purification, *E. coli* DH10B strain was transformed with expression vectors encoding Cas12m and their respective minimal CRISPR region (pTK205-208, pTK213-214, pTK218-219; Supplementary Table S4). The cells were grown in LB broth supplemented with ampicillin (100 μg/ml) at 37°C until OD_600_ reached 0.8. The protein expression was induced with 0.2% l-arabinose and the cells were grown for additional 16 h at 16°C. Cells were pelleted by centrifugation, resuspended in 20 mM Tris–HCl (pH 8.0 at 25°C), 100 mM NaCl, 5 mM 2-mercaptoethanol, 25 mM imidazole, 5% (v/v) glycerol and 1× Halt^TM^ Protease Inhibitor Cocktail (Thermo Fisher Scientific) containing buffer and disrupted by sonication. Cell debris was removed by centrifugation and the remaining supernatant was loaded on Ni^2+^-charged HiTrap chelating HP column (GE Healthcare). The Cas12m RNP complexes were eluted by increasing imidazole concentration from 25 mM to 500 mM in 20 mM Tris–HCl (pH 8.0 at 25°C), 100 mM NaCl, 5 mM 2-mercaptoethanol and 5% (v/v) glycerol buffer. The collected fractions containing Cas12m RNP were dialyzed against 20 mM Tris–HCl (pH 8.0 at 25°C), 100 mM NaCl, 2 mM DTT, and 50% (v/v) glycerol buffer and stored at −20°C.

### Extraction and sequencing of Cas12m bound RNA species

Cas12m RNP samples (100 μl) were treated with 5 μl (20 mg/ml) of Proteinase K (Thermo Fisher Scientific) for 45 min at 37°C in 1 ml of 10 mM Tris–HCl (pH 7.5 at 37°C), 100 mM NaCl, 5 mM MgCl_2_, 1 mM DTT and 1 mM EDTA buffer. Then 2.5 μl (1 U/μl) of DNase I (Thermo Fisher Scientific) was added and the samples were incubated for additional 30 min at 37°C. RNA was purified using GeneJET RNA Cleanup and Concentration Micro Kit (Thermo Fisher Scientific). The extracted RNA (1 μg) was treated with RiboCop rRNA Depletion Kit (Lexogen) and end-repaired with T4 Polynucleotide Kinase (20 U) in 1× reaction buffer A (Thermo Fisher Scientific) supplemented with 1 mM ATP (Thermo Fisher Scientific) for 1 h at 37°C. The reaction products were purified using RNA Clean & Concentrator-5 kit (Zymo Research) and 11–36 ng of RNA was used for library generation with the Small RNA-Seq Library Prep Kit (Lexogen). The obtained libraries were quantified by Qubit 4 Fluorometer (Thermo Fisher Scientific), pooled in an equimolar ratio, and paired-end sequenced (2 × 75 bp) using the MiniSeq High Output Reagent Kit, 150 cycles (Illumina) on a MiniSeq System (Illumina).

The adapter sequences and paired-end reads shorter than 15 bp were filtered using Cutadapt (v2.8) (36). The remaining reads were mapped to the corresponding Cas12m and CRISPR expression plasmids (pTK205-208, pTK213-214, pTK218-219; Supplementary Table S4) using BWA-MEM (v0.7.17-r1188) (37). Paired-end reads with mapping Phred score <30 were filtered out with SAMtools (v1.10) (38). Custom Python script (39) was used to extract and visualize entire RNA sequences (≤65 nt) from paired-end alignments.

### RNA synthesis

Templates for *in vitro* transcription of Cas12m pre-crRNAs were generated by PCR of respective Cas12m systems minimal CRISPR region with addition of a T7 promoter sequence at the 5′-end. Similarly, templates for *in vitro* transcription of RNA targets and crRNAs were generated by PCR of respective oligo duplexes. Obtained RNA templates were transcribed using TranscriptAid T7 High Yield Transcription Kit (Thermo Fisher Scientific) and purified using GeneJET RNA Cleanup and Concentration Kit (Thermo Fisher Scientific). Sequences of the RNAs used in this study are available in Supplementary Table S5.

### pre-crRNA processing assays

pre-crRNA processing was verified using *in vitro* synthesized putative pre-crRNAs and purified Cas12m proteins. Reaction mixtures contained 500 nM pre-crRNA, 2000 nM (or as indicated) Cas12m RNP complex in 10 mM Tris–HCl (pH 7.5 at 37°C), 1 mM EDTA, 1 mM DTT, 100 mM NaCl and 10 mM MgCl_2_ buffer. Reactions were incubated at 37°C for 1 h, followed by protein inactivation with the addition of Proteinase K (final conc. 1 mg/ml) and incubation at 50°C for 10 min. The final quench of the reaction was performed by adding EDTA (15 mM final conc.) and 2× RNA loading dye (Thermo Fisher Scientific), and incubating at 75°C for 10 min. Reaction products were resolved using denaturing urea-PAGE (15% acrylamide:bis-acrylamide 29:1, 8.5 M urea, 0.5× TBE) gel stained with SYBR Gold Nucleic Acid Stain (Invitrogen) and imaged on an Amersham Typhoon phosphorimager (GE Healthcare).

### Cell-free Cas12m RNP complex production

Cell-free Cas12m RNP complexes were obtained using PURExpress *In Vitro* Protein Synthesis Kit (NEB) cell-free *in vitro* transcription/translation (IVTT) system. DNA templates for Cas12m production were generated by PCR from the pBAD expression vectors encoding Cas12m proteins (Twist Biosciences, pTK205-208, pTK213-214, pTK218-219; Supplementary Table S4). crRNAs targeting 20 bp sequence in the plasmid library (pTZ57; Supplementary Table S4) adjacent to a 7 bp (7N) randomized PAM (protospacer adjacent motif) sequence (40,41) were assembled by PCR from oligonucleotides (Supplementary Table S6). IVTT reactions were carried out from Cas12m and crRNA encoding DNA constructs (125 ng each) in 12.5 μl IVTT reaction mixture for 4 h at 37°C. Crude mixtures of such prepared RNP complexes later were used for the following dsDNA cleavage and binding activity determination assays.

### dsDNA cleavage activity determination using plasmid PAM library

To test DNA cleavage activity of Cas12m RNP complexes, the previously developed dsDNA cleavage assay using 7N PAM plasmid library was adopted (7,40,41) with the following modifications. 5 μl of IVTT-prepared crude reaction mixture with Cas12m RNP complex were incubated with 500 ng of pTZ57 7N PAM library in 50 μl reaction buffer containing 10 mM Tris–HCl (pH 7.5 at 37°C), 100 mM NaCl, 1 mM DTT and 10 mM MgCl_2_ for 1 h at 37°C. After incubation, DNA ends were repaired by adding 0.5 μl (2.5 U) of T4 DNA polymerase (Thermo Fisher Scientific), 0.5 μl of 10 mM dNTP mix (Thermo Fisher Scientific) followed by incubation for 20 min at 11°C and 10 min at 75°C. Next, 3′-dA overhangs were added by incubating the mixture with 0.5 μl (2.5 U) of DreamTaq polymerase (Thermo Fisher Scientific) and 0.5 μl of 10 mM dATP (Thermo Fisher Scientific) for 30 min at 72°C. RNA was degraded by incubating for 15 min at 37°C with 0.5 μl of 10 mg/ml of RNase A (Thermo Fisher Scientific), followed by DNA purification using GeneJET PCR Purification Kit (Thermo Fisher Scientific). Next, 100 ng of purified DNA products were ligated with 100 ng of dsDNA adapter containing a 3′-dT overhang (Supplementary Table S6) for 1 h at 22°C using 1 μl (5 U) of T4 DNA ligase (Thermo Fisher Scientific) in 25 μl reaction volume.

The ligation products (2 μl) were PCR amplified with Phusion Hot Start II DNA polymerase (Thermo Fisher Scientific) using TK-737 and an equimolar mixture of TK-735, 765–771 oligonucleotides (Supplementary Table S7) for 17 cycles in 20 μl reaction volume. In the second round of PCR P5 and P7 adapters for Illumina sequencing were added using the Lexogen PCR Add on Kit (Lexogen) with the i7 6 nt Index Set (Lexogen). For this 3 μl of the first round PCR products were used as a template in 30 μl total reaction volume for 17 cycles. Indexed DNA samples were pooled in an equimolar ratio, gel purified with GeneJET Gel Purification Kit (Thermo Fisher Scientific), and quantified by Qubit 4 Fluorometer (Thermo Fisher Scientific). Lastly, samples were single-end sequenced (1 × 150 bp) using the MiniSeq High Output Reagent Kit, 150 cycles (Illumina) on a MiniSeq System (Illumina). dsDNA cleavage activity was evaluated by examining the adapter ligation at the targeted sequence in the plasmid library. The reads containing the adapter ligated at the 0–30 bp target positions next to the 7N region were counted using a custom Python script (39).

### dsDNA binding activity determination using plasmid PAM library

To test the DNA binding activity of Cas12m RNP complexes, the previously developed 7N PAM plasmid library-based dsDNA cleavage assay was modified to include treatment with restriction enzyme, similar to the previously developed assay (42). Briefly, 2 μl of IVTT-reaction mixture with Cas12m RNP complex were incubated with 40 ng of 7N PAM library in 20 μl reaction buffer containing 10 mM Tris–HCl (pH 7.5 at 37°C), 100 mM NaCl, 1 mM DTT and 10 mM MgCl_2_ for 1 h at 37°C. After incubation, the samples were diluted 10 times, and 40 μl of each sample was further incubated with 1 μl of FastDigest Eam1105I (Thermo Fisher Scientific) for 1 h at 37°C, followed by additional incubation for 20 min at 65°C. Next, samples were treated with 1 μl (20 mg/ml) of Proteinase K (Thermo Fisher Scientific) for 30 min at 50°C. RNA was removed by incubating with 1 μl of 10 mg/ml RNase A (Thermo Fisher Scientific) for 15 min at 37°C and for 5 min at 95°C. The resulting reaction mixture of PAM plasmid library (5 μl) was PCR amplified with Phusion Hot Start II DNA polymerase (Thermo Fisher Scientific) using TK-737 and an equimolar mixture of TK-736, 772–778 oligonucleotides (Supplementary Table S7) for 25 cycles in 50 μl reaction volume and purified using the GeneJET PCR Purification Kit (Thermo Fisher Scientific). In the second round of PCR P5 and P7 adapters for Illumina sequencing were added using the Lexogen PCR Add on Kit (Lexogen) with the i7 6 nt Index Set (Lexogen). 2 ng of the products from the first round of PCR were used as a template in 30 μl total reaction volume for 25 cycles. Indexed DNA samples were pooled in an equimolar ratio, gel purified with GeneJET Gel Purification Kit (Thermo Fisher Scientific), and quantified by Qubit 4 Fluorometer (Thermo Fisher Scientific). Lastly, samples were single-end sequenced (1 × 150 bp) using the MiniSeq High Output Reagent Kit, 150 cycles (Illumina) on a MiniSeq System (Illumina). The obtained sequences were used for PAM extraction, frequency calculation, and normalization to the original 7N PAM library to account for inherent biases. To reduce the influence of background noise resulting from the incomplete cleavage of all available targets by Eam1105I, only PAM sequences with >5-fold enrichment compared to the sequenced initial library were extracted and represented in WebLogo format (43). All sequence manipulations and analyses were performed using a custom Python script (39).

### RNP complex assembly

The purified Cas12m protein (1 μM) was combined with its corresponding crRNA in 1:1 molar ratio in complex assembly buffer (10 mM Tris–HCl (pH 7.5 at 37°C), 100 mM NaCl, 1 mM EDTA, 1 mM DTT and 10 mM MgCl_2_) and allowed to incubate for 30 min at 37°C.

### DNA substrate generation

The 5′-ends of oligonucleotides were first radiolabeled using T4 PNK (Thermo Fisher Scientific) and [γ-^32^P]ATP (PerkinElmer). The dsDNA substrates were generated by annealing two oligonucleotides with complementary sequences, one of which had a radioactive label introduced at the 5′-end. Annealing was performed at 95°C followed by a slow cooling to room temperature. The sequences of the DNA target substrates are provided in Supplementary Table S6.

### DNA cleavage assay

Reactions with oligo duplexes or ssDNA oligonucleotides were carried out by mixing labeled DNA samples with preassembled Cas12m or Cas12a RNP complexes and incubating for 1 h at 37°C. Reaction mixtures contained 1 nM labeled oligo duplex, 100 nM Cas12 RNP complex, and 10 mM Tris–HCl (pH 7.5 at 37°C), 1 mM EDTA, 1 mM DTT, 100 mM NaCl and 10 mM MgCl_2_ in a final volume of 50 μl. Aliquots of 6 μl were removed from the reaction mixture and quenched with 10 μl of loading dye (95% (v/v) formamide, 0.01% Bromophenol Blue and 25 mM EDTA). Reaction products were analyzed by denaturing gel electrophoresis (20% polyacrylamide containing 8.5 M urea in 0.5× TBE buffer), followed by gels being dried and visualized by phosphor imaging.

### RNA substrate generation

The 5′-ends of transcribed target RNA molecules were first dephosphorylated using FastAP (Thermo Fisher Scientific) and then radiolabeled using T4 PNK (Thermo Fisher Scientific) and [γ-^32^P]ATP (PerkinElmer). The sequences of RNA targets are provided in Supplementary Table S5.

### RNA cleavage assay

Reactions were carried out by mixing labeled RNA samples with preassembled Cas12m or Cas12a RNP complex and incubating for 1 h at 37°C. Reaction mixtures contained 1 nM labeled RNA, 100 nM Cas12m RNP complex, and 10 mM Tris–HCl (pH 7.5 at 37°C), 1 mM EDTA, 1 mM DTT, 100 mM NaCl and 10 mM MgCl_2_ in a 20 μl final volume. Aliquots of 10 μl were removed from the reaction mixture and quenched with 10 μl of 2× RNA gel loading dye (Thermo Fisher Scientific). Reaction products were analyzed by denaturing gel electrophoresis (15% polyacrylamide containing 8.5 M urea in 0.5× TBE buffer), followed by drying gels and visualizing by phosphor imaging.

### Trans-cleavage assay

Target-activated trans-cleavage activity was measured by performing the fluorophore quencher-labeled reporter assays. Reaction mixtures contained 100 nM preassembled Cas12m RNP complex, 25 nM ssDNA or dsDNA activator, and 100 nM DNaseAlert or RNaseAlert reporters (IDT), respectively. Activator dsDNA duplex was prepared by annealing 1.15-fold molar excess of the non-target strand to the target strand. Reactions were performed in 10 mM Tris–HCl (pH 7.5 at 37°C), 1 mM EDTA, 1 mM DTT, 100 mM NaCl and 10 mM MgCl_2_ buffer in a 50 μl final volume. Reactions were carried out in ClarioStar fluorescence plate-reader (BMG Labtech) for 2h at 37°C, with fluorescence measurements taken every 2 min (DNaseAlert reporter – excitation filter: 533 nm/8 bandpass, emission filter: 559 nm/8 bandpass; RNaseAlert reporter – excitation filter: 484 nm/20 bandpass, emission filter: 529 nm/20 bandpass). The sequences of the DNA activators are listed in Supplementary Table S6.

### Bio-layer interferometry spectroscopy (BLI)

The BLI experiments were performed using an Octet K2 system (Sartorius). The dsDNA substrates were generated by annealing two complementary oligonucleotides, one of which contained biotin at the 5′-end (Supplementary Table S6). Octet High Precision Streptavidin 2.0 (SAX2) Biosensors (Sartorius) were hydrated for 1 h at room temperature in 10 mM Tris–HCl (pH 7.5 at 37°C), 1 mM EDTA, 1 mM DTT, 100 mM NaCl and 10 mM MgCl_2_ incubation buffer supplemented with 0.01% Triton X-100. After a baseline step of 300 s, the SAX2 biosensors were incubated in 25 nM biotin-modified DNA solution for 60 s, while rotating the plate at 1000 rpm. Unbound dsDNA was washed in the incubation buffer for 180 s. The surface of the biosensors was neutralised by incubation in 10 μg/ml biocytin for 180 s, followed by an additional baseline step in the incubation buffer for 120 s to allow for signal stabilization. The biosensors were transferred to the wells containing 50 nM GoCas12m RNP complex to monitor complex association with DNA for 30 min. Finally, the sensors were transferred to the incubation buffer to monitor dissociation of the complex for 2 h. A biosensor without immobilized DNA was used as a reference. The reference signal was subtracted from the signal obtained with a sensor containing immobilized DNA. Assays were performed at 37°C in biological triplicates.

### DNA substrates for magnetic tweezers

The 3800 bp long DNA construct for magnetic tweezers experiments was prepared by PCR amplifying a linear 2600 bp long DNA fragment from pGB108 (Supplementary Table S4) containing the target site using the JMM_For_NotI and JMM_Rev_HindIII primers (Supplementary Table S7). The fragment was digested at its ends with NotI and HindIII for which corresponding sites were introduced in the primer sequences. Subsequently, the fragment was ligated at either end to ∼600 bp PCR fragments containing multiple biotin (HindIII site) or digoxigenin (NotI site) modifications (44).

### Magnetic tweezers experiments

The measurements were performed in a custom-built magnetic tweezers setup that allows for GPU-assisted real-time measurements of the DNA length as described before (45). The DNA constructs were bound on their biotinylated end to streptavidin-coated 1 μm-diameter magnetic beads (MyOne, Invitrogen) and flushed into a flowcell allowing the anchoring of the digoxigenin-modified end to the anti-digoxigenin on the glass surface. After washing unbound beads, DNA-tethered beads were selected by applying force using a pair of magnets. From tracking the axial position of the magnetic beads with respect to a reference bead using a camera (Mikrotron EoSens), the length of the individual DNA molecules was determined and the applied forces were calibrated (46). During the experiments, the applied force was kept constant at 0.3 pN. Supercoiling of DNA was achieved by turning the magnets between −10 and 10 turns. Time trajectories of the DNA length were recorded at 120 Hz and smoothed with a sliding average to 2 Hz. Then, 5 nM GoCas12m RNP in measurement buffer (10 mM Tris–HCl (pH 7.5), 100 mM NaCl, 1 mM EDTA, 1 mM DTT, 10 mM MgCl_2_ and 1 mg/ml BSA) was added. After protein addition, DNA length changes were monitored in real time. Successful formation of an R-loop was confirmed by a shift of the characteristic DNA rotation curve towards negative turns.

### Plasmid interference assay

Plasmid interference assays were performed in the *E. coli* DH10B strain bearing Cas12 (pTK147, pRZ169 and pTK200, pTK203, pTK216-217) and crRNA guide sequence (pRZ166-168, pRZ170-173, pRZ184-188 and pRZ194-201) containing plasmids. The cells were grown at 37°C overnight, diluted 100× in LB medium, and additionally grown to OD_600_ of ∼0.5–0.6. Following that, a third chemical transformation was performed with 250 ng of target encoding plasmids (pKP8 for pRZ169, pTK147, pTK200, pKP9 for pTK203 and pKP11 for pTK216-217). The co-transformed cells were further diluted by serial 10× fold dilutions and grown at 37°C overnight on plates containing l-arabinose (0.2%), glucose (1%), carbenicillin (100 μg/ml), chloramphenicol (15 μg/ml) and kanamycin (100 μg/ml). The efficiency of interference calculations for a given condition was performed by normalizing the mean of colony-forming units (CFU) for a condition to the mean CFU of a non-targeting control: mean(CFU_condition)/mean(CFU_negative_control). All interference assays were performed in biological triplicates. Descriptions and links to plasmid sequences can be found in Supplementary Table S4. Target sequences can be found in Supplementary Table S8.

### GFP silencing assay

Similarly to plasmid interference assays, silencing assays were performed in *E. coli* DH10B strain bearing Cas12 (pTK147, pRZ169 and pTK200) and crRNA (pRZ166-167, pGB109-120) encoding plasmids. The cells were grown at 37°C overnight, diluted 40× in LB medium, and additionally grown to OD_600_ of ∼0.5–0.6. A third chemical transformation was performed with 250 ng of target sequences containing plasmid (pGB108) which was obtained by cloning superfolder GFP (sfGFP) encoding biobrick (gift from iGEM, K515105) into pSG4K5 (gift from Xiao Wang, Addgene plasmid #74492). The co-transformed cells were further diluted by serial 10× fold dilutions and grown at 37°C for 24 h on plates containing L-arabinose (0.2%), glucose (1%), streptomycin (50 μg/ml), carbenicillin (100 μg/ml), chloramphenicol (25 μg/ml) and kanamycin (50 μg/ml). Plates were visualized by fluorescence imaging (488 nm laser; blue filter; Amersham Typhoon scanner) and white light imaging (white light epi-illumination; Uvitec Firereader V10). GFP silencing assays were performed in biological triplicates. Descriptions and links to plasmid sequences can be found in Supplementary Table S4. Target sequences can be found in Supplementary Table S8.

### Bacteriophage plaque assay

In this study, ER2267, DH10B and BL21 (DE3) *E. coli* strains were used for phages M13mp18, λvir and T4, and VpaE1 respectively. Bacteriophage assays were conducted following a modified double agar overlay protocol. Different *E. coli* strains containing both Cas12 effector (pTK147, pRZ169 and pTK200) and crRNA (pRZ166-167, pGB085-100) encoding plasmids were grown overnight at 37°C, 200 rpm. 1 ml of saturated overnight culture was mixed with 4 ml molten 0.75% (w/v) agar LB for the top layer, spread onto a corresponding LB agar plate (1.50% (w/v) agar, 1% (w/v) tryptone, 0.5% (w/v) yeast extract, 0.5% (w/v) NaCl, 100 μg/ml carbenicillin, 25 μg/ml chloramphenicol, different l-arabinose concentrations (0.01% for VpaE1 and 0.001% for the rest)) and allowed to completely solidify. The selected phages were further diluted by serial 10× fold dilutions with LB (with 5 mM MgCl_2_ addition for λvir). 2 μl of each dilution were spotted onto the top agar and grown for 16–20 h at 37°C. After overnight incubation, the effect was evaluated by counting plaque-forming units (PFUs). If PFUs were not enumerable, but clearings were observed at high phage concentrations, the most concentrated dilution at which no plaques/clearings were observed was counted as 1 PFU. Efficiency of plating (EOP) calculations for a given condition were performed by normalizing the mean of PFU for a condition to the mean PFU of a non-targeting control: mean(PFU_condition)/mean(PFU_negative_control). Plaque assays were performed in biological triplicates. Descriptions and links to plasmid sequences can be found in Supplementary Table S4. Target sequences can be found in Supplementary Table S8.

### Cryo-EM sample preparation

The GoCas12m-crRNA-DNA complex was reconstituted by mixing purified GoCas12m and its crRNA with dual-end oligoduplex DNA at a molar ratio of 2:2:1. First, the purified GoCas12m protein was combined with its corresponding crRNA and allowed to incubate for 30 min at room temperature. Next, dual-end oligoduplex DNA was added with additional incubation for 30 min at room temperature. Oligoduplex DNA was assembled by annealing target sequences containing oligonucleotides (Supplementary Table S6). Reaction mixtures contained 40 mM Tris–HCl (pH 8.0 at 37°C), 150 mM NaCl, 10 mM MgCl_2_ and 1 mM 2-mercaptoethanol buffer. Lastly, the complex solution (roughly 10 μM, 3 μl) was applied to freshly glow-discharged copper 300 mesh R1.2/1.3 holey carbon grids (Quantifoil), in a Vitrobot Mark IV (FEI) at 4°C with a waiting time of 0 s and a blotting time of 5 s under 95% humidity conditions. The grids were plunge-frozen in liquid ethane cooled at liquid nitrogen temperature.

### Electron microscopy data collection and processing

The cryo-EM data for the GoCas12m-crRNA-DNA ternary complex were collected using a Glacios microscope (Thermo Fisher Scientific), running at 200 kV and equipped with a Falcon 3EC Direct Electron Detector in the electron counting mode (Vilnius University, Lithuania). Images were recorded with EPU (v.3.2) at a nominal magnification of ×92 000, corresponding to a calibrated pixel size of 1.10 Å per pixel, using an exposure of 0.80 e/Å^2^ s^−1^, in 30 frames and a final dose of 29.7 e/Å^2^, over a defocus range of −1.0 to −2.0 μm. Patch motion correction, CTF estimation, micrograph curation, blob picking and particle extraction were performed in real-time in CryoSPARC Live (v.4.2.1) (47,48). Further data processing was performed using standard CryoSPARC (v.4.2.1) (47,48). The 1 559 728 particles of GoCas12m ternary complex were extracted (box size 200 pixels) from 1146 accepted micrographs. After 2D classification, the selected particles (492 787) were subjected to heterogeneous refinement using two volumes obtained from Ab-Initio job. Class 0 possessing higher FSC resolution (3.83 Å, 325 306 pct) was further subjected to 3D classification to five classes. After 3D classification, particles from two selected classes (204 822) were used for the final reconstruction using local refinement.

The global resolution and sphericity values for all reconstructions were estimated using 3DFSC v.3.0 software (49) according to the Fourier shell correlation of 0.143 criterion (50). The local resolution was estimated in CryoSPARC (v.4.3.0) (47,48).

### Model building and validation

The initial protein model was generated using AlphaFold (51) under the ColabFold (52) framework using default parameters and MMseqs2 to search for homologues into the ColabFold database, and manually modified using Coot (v.0.9.8.1) (53) against the map sharpened using phenix.auto_sharpen (v.1.20.1–4487) (54). crRNA and DNA were built manually. Model refinement was performed using phenix.real_space_refine (v.1.20.1–4903) (54). The final model of the ternary complex covers 1–604 protein residues and –31 to 20 nucleotides of the crRNA. The statistics of the 3D reconstruction and model refinement are summarized in Supplementary Table S9. The molecular graphics figures were prepared with ChimeraX (v.1.5) (55) and PyMOL (v.2.3.0) (The PyMOL Molecular Graphics System, Schrödinger, LLC).

### Cloning of Cas12 base editors expression vectors

To obtain eukaryotic GoABE expression plasmid (pTK225), *enAsCas12a* sequence in *enAsABE* encoding plasmid (pTK221; gift from David R. Liu, Addgene plasmid #138506) was replaced with human codon-optimized *goCas12m* gene (Twist Bioscience) using BamHI and PaeI restriction enzymes (Thermo Fisher Scientific). crRNA encoding constructs were cloned under U6 promoter using Gibson assembly to obtain pBD1-2 and pGB125-126 plasmids. For base editing assay in *E. coli* cells, *goABE* or *enAsABE* genes (obtained from pTK225 or pTK221) and respective crRNA encoding sequences with J23119 promoter were cloned into pETDuet1 (MilliporeSigma) expression vector with XbaI/NotI and Bsp1407I/XhoI (Thermo Fisher Scientific) restriction enzymes, respectively (pGB129-132). Descriptions and links to plasmid sequences can be found in Supplementary Table S4.

### Chloramphenicol resistance recovery assay

Chloramphenicol resistance recovery assays were performed in *E. coli* BW25141 (λDE3) strain (gift from David R. Edgell) bearing GoABE or enAsABE and crRNA guide sequences encoding plasmids (pGB129-132). The cells were transformed with an inactivated chloramphenicol resistance gene (*cmR*) containing plasmid (pGB121), obtained from pACYC184 (New England Biolabs) vector by QuickChange mutagenesis to introduce premature stop codon into *cmR* gene. After transformation, individual colonies were picked and grown at 37°C overnight in LB supplemented with tetracycline (10 μg/ml) and carbenicillin (100 μg/ml). Next, the cells were diluted 100× in LB medium and grown to OD_600_ of ∼0.4. The expression of ABEs was induced with 0.5 mM of IPTG and the cells were grown overnight at 37°C, 200 rpm. After overnight growth, the cells were further diluted by serial 10× fold dilutions and grown at 37°C overnight on plates containing carbenicillin (100 μg/ml), chloramphenicol (25 μg/ml), and tetracycline (10 μg/ml). Base editing activity in the recovered cells was evaluated by Sanger sequencing. The assays were performed in biological triplicates. Descriptions and links to plasmid sequences can be found in Supplementary Table S4. Target sequences can be found in Supplementary Table S8.

### HEK293T cultivation

HEK293T cells obtained from ATCC (catalog number CRL-3216) were cultivated in DMEM medium (Gibco) supplemented with 10% FBS (Gibco), penicillin (100 U/ml) and streptomycin (100 μg/ml), and grown at 37°C and 5% CO_2_. The cells were transfected at 70–80% confluency and grown for 72 h unless specified otherwise.

### GFP recovery assay

HEK293T cells were seeded at 1.4×10^5^ cells per well in 24-well plates. 24 h after seeding, cells were co-transfected with 400 ng GoABE or enAsABE expression plasmids (pTK225 or pTK221), 200 ng crRNA expression plasmids (pBD1-2 and pGB125-126), and 400 ng inactive *eGFP* encoding plasmid (pGB122) obtained from *eGFP* encoding construct (pRZ174; gift from Toni Cathomen) by QuickChange mutagenesis to introduce premature stop codon, using 2.5 μl TurboFect Transfection Reagent (Thermo Fisher Scientific). The medium was changed at 24 h post-transfection and flow cytometry analysis was performed at 24, 48 and 72 h post-transfection with Partec CyFlow Space flow cytometer (Sysmex Partec, Goerlitz, Germany). Data analysis and visualisation were performed with FlowJo software (FlowJo LLC, Ashland, OR, USA). The initial data of screening has been summarized in Supplementary Table S10. Descriptions and links to plasmid sequences can be found in Supplementary Table S4. Target sequences can be found in Supplementary Table S8.

### Genome base editing assay

HEK293T cells were seeded at 30 000 cells per well in 96-well plates. 24 h after seeding, cells were co-transfected with 150 ng of GoABE or enAsABE expression plasmids (pTK225 or pTK221) and 50 ng of crRNA (pBD1-2, pBD5-6, pBD9-10, pBD17-18 and pBD23-26) encoding plasmids using 0.5 μl Turbofect transfection reagent (Thermo Fisher Scientific). Cells were cultured for 72 h, then washed with 1× PBS (Thermo Fisher Scientific), followed by cell lysis with 30 μl QuickExtract solution (Lucigen). Next, two rounds of PCR were performed: first to amplify the DNA region surrounding each target site, and second to add the sequencing adapters required for Illumina sequencing. Briefly, 1 μl of cell lysate was used in the primary PCR with primers specific to the targeted genomic locus (Supplementary Table S7), in a final volume of 20 μl, using Phusion High-Fidelity DNA Polymerase (Thermo Fisher Scientific). A total of 1.5 μl first round PCR product in a final volume of 30 μl was used as a template for the second round of PCR to index and add P5 and P7 adapters required for Illumina sequencing. Samples were pooled in an equimolar ratio and purified from agarose gel using a GeneJET Gel extraction kit (Thermo Fisher Scientific). Barcoded and purified DNA samples were quantified with Qubit 4 Fluorometer (Thermo Fisher Scientific), analyzed using BioAnalyzer (Agilent), and pair-end sequenced (2 × 75 bp) using the MiniSeq High Output Reagent Kit, 150 cycles (Illumina) on a MiniSeq System (Illumina). Base editing efficiency was analyzed using CRISPResso2 (56). Descriptions and links to plasmid sequences can be found in Supplementary Table S4. Target sequences can be found in Supplementary Table S8.

## Results

### Identification and characterization of subtype V-M (V-U1) CRISPR-Cas12m effectors

**Figure 1.**
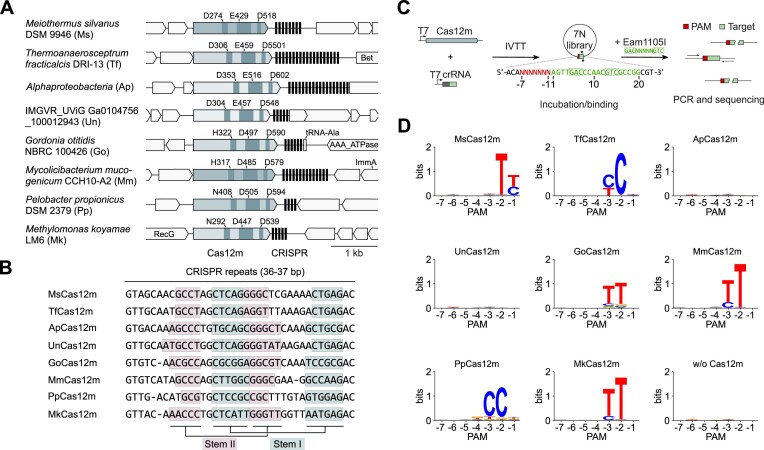
Characterization of subtype V-M CRISPR-Cas12m systems. **(A)** Schematic representation of 8 subtype V-M CRISPR-Cas12m systems. Amino acid residues at the predicted Cas12m RuvC-like active site are indicated above each *cas12m* gene. **(B)** Repeat sequences of the CRISPR-Cas12m systems. Light blue and pink colored boxes indicate predicted stem I and stem II secondary structures. **(C)** Experimental workflow of the dsDNA binding based PAM identification assay. Cas12m and crRNA targeting PAM library were synthesized using an *in vitro* transcription-translation (IVTT) system. The resulting solution containing Cas12m RNP complexes was incubated with the PAM library, followed by restriction enzyme treatment. Uncleaved dsDNA fragments were amplified by PCR and sequenced. **(D)** WebLogo representation of the PAM sequences characterized for Cas12m proteins.

We performed a Protein BLAST search in sequence databases using the V-U1 Cas12m protein from *Gordonia otitidis* (Go) (2,3) as an initial query, which resulted in a non-redundant list of 134 Cas12m family variants identified in diverse bacteria (Supplementary Table S1). For experimental characterization, we selected eight variants with different arrangements of the putative RuvC active site: four variants containing a typical D-E-D triad, two (including MmCas12m) – H-D-D, and two – N-D-D triads (Figure 1A and Supplementary Table S2). Analysis of the repeat sequences from the CRISPR regions in the vicinity of *cas12m* genes showed high sequence conservation between orthologous CRISPR-Cas12m systems and predicted secondary structures within the repeat (Figure 1B), similar to other type V systems (9,12,14).

To experimentally characterize the Cas12m effectors, we first aimed to determine the guide RNA requirements. The Cas12m ribonucleoprotein (RNP) complexes were heterologously expressed in *E. coli* cells from the plasmids harboring the *cas12m* gene and an engineered minimal CRISPR region containing a single spacer. The RNA isolated from the purified RNP complexes was sequenced (Supplementary Figure S1A), revealing enrichment of crRNAs derived from the CRISPR region (Supplementary Figure S1B). Examination of the 5′-crRNA ends suggested processing of the precursor CRISPR RNA (pre-crRNA) within the repeat sequences (Supplementary Figure S2A). Sequence analysis of enriched RNAs derived from regions adjacent to the CRISPR array did not reveal complementarity to the repeat sequences, ruling out the presence of the trans-activating RNAs (tracrRNAs) (8–10,13) and indicating that Cas12m proteins are guided to the DNA target by a single short (50–60 nt) crRNA. The absence of tracrRNA and the observed cleavage within the repeat sequences implied Cas12m-mediated processing of pre-crRNA which was also suggested for MmCas12m (29) and demonstrated for other type V systems (9,12,57). To test this, we incubated the purified *Meiothermus silvanus* (Ms) Cas12m with the *in vitro* transcribed pre-crRNA mimicking RNA (Supplementary Figure S2B). Analysis of the reaction products revealed Mg^2+^- and MsCas12m-dependent cleavage of the pre-crRNA within the repeat sequence. Interestingly, the D274A substitution of the predicted RuvC catalytic site residue had no effect on pre-crRNA processing, indicating that pre-crRNA cleavage is RuvC-independent as previously demonstrated for MmCas12m (28).

### Cas12m effectors bind DNA targets in a PAM-dependent manner

For efficient dsDNA targeting, Cas12 effectors require a protospacer adjacent motif (PAM) next to the DNA sequence recognized by guide RNA (gRNA) (11,14). To test whether Cas12m proteins guided by a single crRNA can recognize and cleave dsDNA in a PAM-dependent manner, we adopted the protospacer/transposon adjacent motif (PAM/TAM) identification assay, previously developed for the characterization of Cas9/Cas12 and TnpB nucleases (11,16,40). Firstly, Cas12m proteins and crRNAs, encoded in PCR-generated DNA fragments, were expressed using a cell-free *in vitro* transcription-translation (IVTT) system (Supplementary Figure S3A). Next, the IVTT mixture with produced Cas12m complexes was incubated with the plasmid library, engineered to contain a 7 bp (7N) randomized region 5′ to the target site covering all possible PAM sequences. The DNA ends that would be generated after target DNA cleavage were repaired with T4 polymerase and used for adapter ligation, PCR amplification, and sequencing. The DNA cleavage activity was assessed by scanning the target sequence for increased frequencies of adapter ligation events, which would indicate the cleavage by Cas12m effectors at the target site. Interestingly, adapter ligation frequencies were similar for all samples, including the negative control containing no Cas12m complex, indicating the absence of dsDNA target cleavage activity of Cas12m effectors (Supplementary Figure S3B).

The lack of detectable dsDNA cleavage for all tested Cas12m variants prompted us to investigate whether selected Cas12m effectors can specifically bind to DNA as was previously shown for the MmCas12m and Cas12c variants (29,58). To test the dsDNA binding activity of Cas12m, the 7N plasmid library was incubated with an aliquot of IVTT mixture containing Cas12m complexes. The DNA binding efficiency evaluated using a restriction enzyme (RE) accessibility assay (Figure 1C). Since the Cas12m target site in the plasmid library contained the Eam1105I RE target sequence, we reasoned that Cas12m RNP binding would prevent the RE cleavage. After PCR amplification and sequencing, we detected that 6 tested Cas12m variants interfered with Eam1105I cleavage at the Cas12m target site flanked by T- and C-rich PAM sequences (Figure 1D), consistent with PAM-dependent dsDNA binding by Cas12m effectors.

To probe DNA binding by Cas12m *in vitro*, we employed the bio-layer interferometry (BLI) assay. Biotinylated dsDNA substrates were loaded onto streptavidin-coated BLI biosensors and then binding and dissociation of GoCas12m to dsDNA were measured. The sensorgrams revealed that GoCas12m binds to DNA in a PAM- and target sequence-dependent manner (Supplementary Figure S4A). The formed complexes are highly stable, as only approximately 50% GoCas12m dissociated during 2 h incubation (this corresponds to a dissociation rate of approx. 0.006 min^−1^, Supplementary Figure S4A). Additionally, we investigated R-loop formation through single-molecule DNA twisting experiments utilizing magnetic tweezers, a well established technique previously used for characterization of other CRISPR-Cas effectors (Supplementary Figure S4B) (59,60). We found that upon addition of 5 nM GoCas12m, rotation curves shifted by ∼2 turns demonstrating that GoCas12m successfully formed R-loops of ∼20 bp that were stable enough to resist positive twist (Supplementary Figure S4C).

In a separate set of experiments, we showed that Ms and GoCas12m effector complexes assembled from purified components do not cleave target-containing DNA or RNA *in vitro*, whereas AsCas12a exhibits robust DNA target cleavage (Supplementary Figure S5A, B). In addition, unlike AsCas12a, the Ms and GoCas12m effectors do not display collateral DNA cleavage activity (Supplementary Figure S5C), which has been previously reported for other Cas12 proteins (8,9,11,61), nor collateral RNA cleavage (Supplementary Figure S5D).

Together, these data reveal an evolutionary conserved PAM-dependent dsDNA binding feature of the cleavage-deficient Cas12m effectors.

### CRISPR-Cas12m provides plasmid DNA interference in *E. coli*

**Figure 2.**
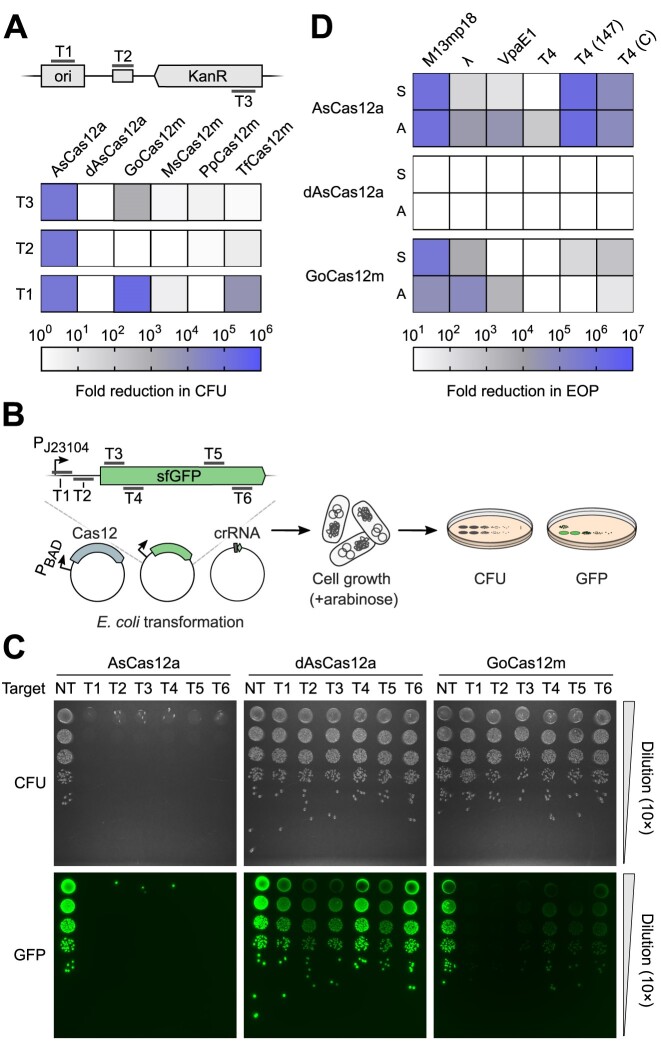
CRISPR-Cas12m activity in *E. coli* cells. **(A)** Plasmid DNA interference assay in *E. coli*. To assess transformation efficiency, each *E. coli* transformant sample was serially diluted (10×) and grown on Kn supplemented media at 37°C overnight. Effective DNA interference of the targeted plasmid resulted in a reduction in colony forming units (CFU). Data are presented as mean (*n* = 3). KanR – kanamycin resistance, ori – origin of replication, dAsCas12a – AsCas12a RuvC active site mutant (D908A). **(B)** Experimental workflow of the superfolder GFP (sfGFP) fluorescence interference assay in *E. coli*. **(C)** sfGFP fluorescence interference experiment in *E. coli*. To assess transformation efficiency and sfGFP expression, each *E. coli* transformant sample was serially diluted (10×) and grown overnight at 37°C on the arabinose-supplemented media. Effective DNA binding by dAsCas12a and GoCas12m resulted in reduction of sfGFP fluorescence while CFU remained unchanged. NT – non-targeting control. **(D)** Bacteriophage plaque formation assay in *E. coli*. To assess the efficiency of plating (EOP), phages were serially diluted (10×) and spotted onto lawns of *E. coli* expressing AsCas12a, dAsCas12a or GoCas12m. Effective defense against phage infection resulted in reduction of plaque-forming units. Data are presented as mean (n = 3). S and A indicate targeted sense and antisense DNA strands, respectively.

The demonstration of PAM-dependent dsDNA binding by Cas12m effectors raised the question if type V-M CRISPR-Cas can function as a defence system and confer resistance to MGEs by targeted DNA binding rather than cleavage. We selected four Cas12m variants with different RuvC active site residue configurations and tested their ability to interfere with plasmid DNA transformation. Briefly, *E. coli* cells expressing Cas12m and crRNA were transformed with a plasmid containing the corresponding PAM and target sequences either in the origin of replication (ori), the plasmid backbone, or the kanamycin resistance (KanR) gene (Figure 2A) and grown on kanamycin (Kn)-supplemented agar plates. The transformation efficiency was assessed by counting colony forming units (CFU) after serial dilution of transformants and represented as the fold change in CFU when compared to the non-targeting control. *Acidaminococcus sp*. Cas12a (AsCas12a) nuclease and catalytically-dead dAsCas12a variants (14) were included for reference. As expected, the AsCas12a nuclease provided efficient plasmid interference in *E. coli* for all tested targets, while cleavage-deficient dAsCas12a had no effect on transformation efficiency when compared to the non-targeting control (Figure 2A and Supplementary Figure 6A). In contrast to dAsCas12a, plasmid interference was observed for the TfCas12m and GoCas12m variants. The functional targets for TfCas12m and GoCas12m were located in the ori, and KanR gene for GoCas12m, indicating that Cas12m mediates plasmid interference by hindering plasmid replication initiation or acting as a roadblock for transcription of an essential selection marker gene.

To confirm that binding by Cas12m can block transcription, we adopted an assay based on superfolder (sf) GFP expression (Figure 2B). Briefly, the *E. coli* cells expressing GoCas12m, dAsCas12a or AsCas12a complex components targeting *sfGFP* gene were transformed with the sfGFP expression plasmid, and transformation efficiency and sfGFP fluorescence levels in the cells were assessed. Serial dilution of the transformants revealed plasmid interference in cells containing AsCas12a. In contrast, the cells expressing GoCas12m and dAsCas12a showed no reduction in transformation efficiency compared to non-targeting controls (Figure 2C), supporting the observation that nuclease-deficient Cas12m effectors provide the interference activity only when targeting essential genes (Figure 2A). However, the sfGFP fluorescence readout revealed reduced expression of *sfGFP* when targeted by GoCas12m and dAsCas12a, both within the promoter or the coding region of *sfGFP* (Figure 2C), implying binding-based transcription inhibition.

### CRISPR-Cas12m protects *E. coli* from bacteriophages

To test whether Cas12m effectors could also confer immunity against bacteriophages, we performed a phage plaque assay using GoCas12m. Complexes were directed to either sense or antisense strands within the essential genes of M13mp18, lambda-vir (λ), VpaE1, and T4 phages. To assay the influence of epigenetic modifications on effector binding, T4 phage variants containing glycosylated (WT), 5-hydroxymethylated (147) or unmodified (C) cytosines in their genome were used. The efficiency of plating (EOP) was evaluated and results were expressed as fold change in EOP compared to non-targeted Cas12 controls (Figure 2D and Supplementary Figure S6B). Similarly to the results of the plasmid transformation assay, the AsCas12a effector provided a reduction in plaque formation with variable efficiency for almost all phages tested except T4 (WT) (Figure 2D). The limited AsCas12a immunity against T4 (WT) is most likely due to the naturally occurring glycosylation of cytosines, as the T4 phage variants containing hydroxymethyl or unmodified cytosines were efficiently targeted. Consistently, similar behavior against T4 was also observed for the Cas12a variant from *Lachnospiraceae bacterium* (62). In contrast to nuclease-deficient dAsCas12a, which was unable to prevent plaque formation for all phage variants tested, GoCas12m efficiently protected *E. coli* against M13mp18, λ and unmodified cytosine-containing T4 phages (Figure 2D). Furthermore, GoCas12m also provided anti-phage immunity function against VpaE1 and 5-hydroxymethylated T4 phages when targeting antisense and sense strands of the phage genomic DNA, respectively (Figure 2D).

Overall, these results suggest that compact Cas12m effectors provide immunity against invading MGEs through targeted DNA binding implying DNA binding-mediated immunity mechanism of the type V-M CRISPR-Cas system.

### Cryo-EM structure of GoCas12m-crRNA-DNA ternary complex

**Figure 3.**
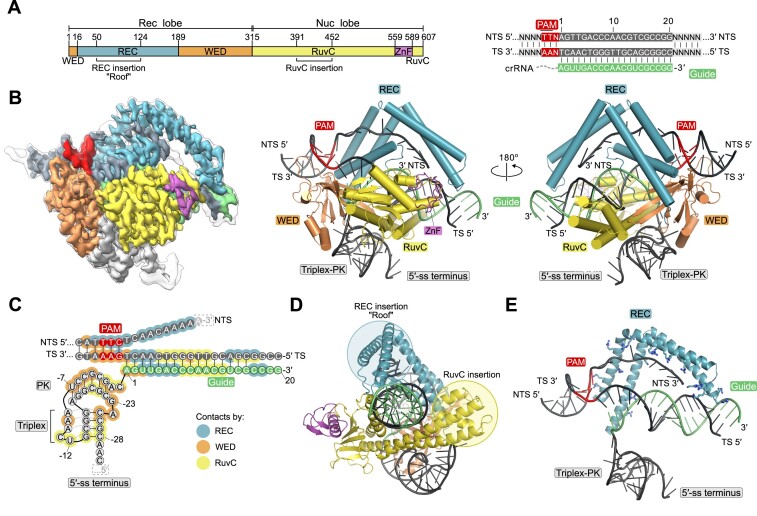
Cryo-EM structure of the GoCas12m-crRNA-DNA ternary complex. **(A)** Schematic representation of the GoCas12m domain organization, crRNA and target DNA. **(B)** Cryo-EM maps (left) and cartoon representations (middle and right) of the GoCas12m-crRNA-DNA ternary complex. Both sharpened (coloured) and unsharpened (black outline) cryo-EM maps are shown. **(C)** Schematic representation of the bound nucleic acids and protein contacts in the complex. Dashed rectangles indicate unresolved parts of the crRNA or DNA. **(D)** Representation of the REC (‘Roof’) and RuvC insertions in GoCas12m. **(E)** View of the interactions between positive amino acid residues in the REC ‘Roof’ and the target DNA. TS – target strand, NTS – non-target strand and PK – pseudoknot.

The DNA binding-based interference distinguishes Cas12m effectors from other Cas12 proteins and the closely related TnpB endonucleases, which are capable of DNA cleavage (9,11–16). To better understand the molecular mechanism of DNA silencing by Cas12m effectors, we have determined the structure of GoCas12m bound to crRNA and target DNA using cryogenic-electron microscopy (cryo-EM) (Supplementary Figure S7). The 2.93 Å structure of GoCas12m-crRNA-DNA ternary complex revealed that GoCas12m protein adopts a bi-lobed structure, characteristic to other Cas12/TnpB proteins, consisting of the N-terminal recognition (Rec) and C-terminal nuclease (Nuc) lobes (Figure 3A, B). The Rec lobe contains wedge (WED) and REC domains, while the Nuc lobe includes a RuvC-like domain with an unusual H322-D497-D590 triad at the active site and a Zinc finger (ZnF) domain.

In comparison to TnpB, which comprises the minimal functional core of the Cas12 nuclease family, Cas12m has acquired a long ɑ-helical insertion (termed ‘Roof’) in the REC domain (residues 50–124), a larger ɑ-helical subdomain between the ꞵ11- and ꞵ12-strands of the RuvC domain, and several insertions in the WED domain (Supplementary Figure S8A). Unlike TnpB, which is guided by a long ∼150 nt reRNA (right-end element RNA, which is also named omega RNA, ωRNA) (15,16), GoCas12m uses a much shorter (∼50 nt) crRNA. The ∼30 nt CRISPR repeat-derived GoCas12m crRNA fragment folds into a compact structure consisting of a triplex region and a pseudoknot (PK) (Figure 3C), which is anchored to GoCas12m WED and RuvC domains by hydrogen bond (H-bond) and van der Waals (vdW) contacts (Supplementary Figure S8B). Despite a notable size difference, this crRNA structure is reminiscent of the triplex-PK core of TnpB reRNA (63,64) (Supplementary Figure S9). The positions of additional hairpin elements present in TnpB reRNA within the GoCas12m-crRNA complex are partially occupied by insertions in the GoCas12m WED and RuvC domains.

The 5′-TTN-3′ PAM-containing region of the target dsDNA is accommodated between the REC and WED domains. All four bases of the 5′-TT-3′ dinucleotide are read out through base-specific H-bond and vdW contacts (Supplementary Figure S8B, C). Separation of the target (TS) and non-target (NTS) DNA strands immediately downstream of the PAM is facilitated by the REC domain residues Y134 and N156 that insert between the two strands and stabilize the last unseparated DNA base pair (Supplementary Figure S8D). The guide RNA-TS DNA heteroduplex is enclosed between the Nuc and Rec lobes and is stabilized by multiple interactions between the phosphodiester backbone and residues from the WED and RuvC domains, similar to the smaller TnpB enzyme (Figure 3C and Supplementary Figure S8B). The most prominent feature of the GoCas12m ternary complex is the ‘Roof’, an arginine-rich ɑ-helical REC domain structural element, that makes multiple non-specific contacts with the NTS strand in the PAM-proximal region, and also interacts with 15–18 bp of the guide RNA-TS DNA heteroduplex. Contacts to the PAM-distal end of the RNA-DNA heteroduplex by the ‘Roof’ and the large ɑ-helical insertion of the RuvC domain extend the readout length of the heteroduplex from 12 bp observed in TnpB to 18 bp in GoCas12m (Figure 3D, E, and Supplementary Figure S8A, B). Presumably, these additional structural elements acquired by GoCas12m ensure a more efficient and stringent target DNA binding that enables immunity in the absence of DNA cleavage. Notably, the GoCas12m shares high structural similarity with MmCas12m (PDB: 8HHL, 52% sequence identity, root-mean-square deviation [RMSD] of 1.1 Å for 577 equivalent Cɑ atoms) (Supplementary Figure S10), suggesting a conserved DNA recognition mechanism across the Cas12m protein family.

### Harnessing GoCas12m for adenine base editing in *E. coli* and human cells

Targeted adenine and cytosine base editors (ABEs and CBEs, respectively) emerged as a potentially safer alternative to the genome editing technology that relies on the introduction of double-stranded breaks (DSBs) (65,66). In the typical base editor (BE), the Cas9 nickase or nuclease-inactivated dCas12a effector is fused to an adenine or cytosine deaminase that enables direct A-to-G or C-to-T conversion, respectively, without DSB. However, such BEs cannot be delivered into cells using a single AAV due to the large size of the BE-encoding gene (>4.7 kb). Therefore, taking into consideration the small size and robust DNA silencing activity of GoCas12m in *E. coli* cells, we reasoned that the GoCas12m could be an attractive candidate for engineering compact BEs.

**Figure 4.**
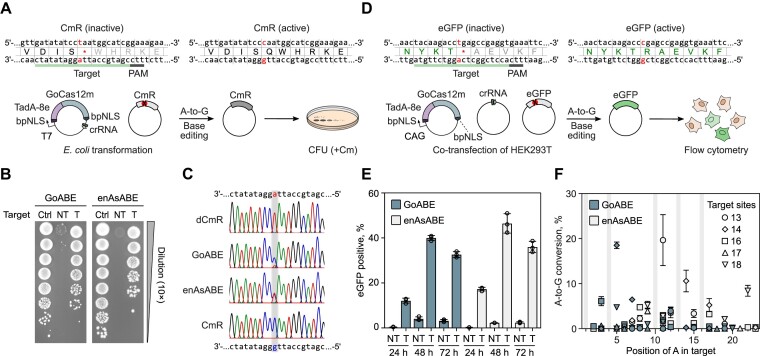
GoABE base editing activity in *E. coli* and human cells. **(A)** Schematic representation of the experimental workflow to detect base editing activity in *E. coli* resulting in chloramphenicol resistance (CmR) gene restoration. **(B)** Plasmid DNA transformation assay in *E. coli*. To assess base editing efficiency, each *E. coli* transformant sample was serially diluted (10×) and grown overnight at 37°C on the Cm supplemented media. Recovery of the colonies indicates successful targeted base editing. As a positive control (Ctrl), *E. coli* were transformed with a plasmid encoding the intact CmR gene. NT and T indicate non-targeting and targeting crRNA constructs, respectively. **(C)** Sanger sequencing of plasmids obtained from recovered *E. coli* colonies. **(D)** Experimental workflow of the enhanced GFP (eGFP) recovery assay performed in HEK293T cells. **(E)** Flow cytometry results of counting eGFP-positive HEK293T cells after 24, 48 and 72 h. Data are presented as mean ± SD (*n* = 3). **(F)** Base editing at endogenous sites in HEK293T cells. Selected DNA target sites are labeled according to Richter *et al.* (67). Gray areas indicate non-A nucleotides at specific positions across all targets. Data are presented as mean ± SD (*n* = 3).

We engineered a GoABE variant by fusing TadA-8e adenine deaminase (67) to the N-terminus of GoCa12m and tested its base editing potential in *E. coli* and human cells. To evaluate GoABE activity in *E. coli*, we used a selection plasmid containing the chloramphenicol resistance (CmR) gene inactivated by a premature stop codon (Figure 4A). GoABE-induced A-to-G conversion within the stop codon, resulting in restoration of chloramphenicol (Cm) resistance, was evaluated by growing *E. coli* cells on Cm supplemented media. We found that *E. coli* cells expressing GoABE with a non-targeting crRNA were not able to form colonies on Cm plates, while cells expressing the GoABE targeting premature stop codon exhibited efficient growth, indicating efficient reversal of inactivating *cmR* gene mutation due to base editing (Figure 4B). Similar results were observed using engineered enAsCas12a (68) fused to TadA-8e (enAsABE), for which adenine base editing activity was previously confirmed (67). Sequencing of the plasmids extracted from single colonies revealed targeted A-to-G editing for both GoABE and enAsABE (Figure 4C).

Next, we probed whether the engineered GoABE is active in human cells. First, we used the reporter plasmid containing a stop codon within the gene encoding enhanced GFP (eGFP) (Figure 4D). After co-transfection of the plasmids encoding GoABE, crRNA, and a reporter into HEK293T cells we performed flow cytometry analysis of cells after 24, 48 and 72 h using enAsABE as a reference. The results revealed an increase in eGFP-positive cells after co-transfection of GoABE and enAsABE with reporter-targeting crRNAs (up to 40% and 47% after 48 h, respectively) when compared to the non-targeting ABE constructs, indicating efficient A-to-G base editing (Figure 4E and Supplementary Figure S11). We next tested whether endogenous genomic sites could be modified using GoABE. We selected five targets that were previously examined for editing with enAsABE (67) and measured A-to-G conversion after 72 h. The results showed that A-to-G editing reached up to 19% and 20% for GoABE and enAsABE, respectively (Figure 4F and Supplementary Figure S12), indicating the efficient base editing activity of GoABE. Interestingly, while the A-to-G conversion levels were similar between the two base editors, the editing windows were rather different (Figure 4F and Supplementary Figure S12). GoABE showed a preference for A-to-G conversion at PAM-proximal positions, which remained inaccessible to enAsABE. Taken together, these data demonstrate that nuclease-deficient Cas12m proteins, as exemplified by GoCas12m, can be adopted as a platform for base editing in *E. coli* and human cells, providing a unique editing window and compact BE size.

## Discussion

In this study, we aimed to characterize a set of compact Cas12m orthologs (∼600 aa) from subtype V-M CRISPR-Cas systems. So far, the only MmCas12m effector (containing an atypical RuvC H-D-D triad) of this family has been characterized revealing PAM-dependent binding of dsDNA (29). We have focused on the set of Cas12m variants that represent the diversity of RuvC active sites observed in Cas12m family and showed that Cas12m orthologs containing D-E-D, H-D-D and N-D-D motifs in the RuvC active site do not exhibit dsDNA nuclease activity, but efficiently bind to dsDNA targets in a PAM-dependent manner (Figure 1C, D). Importantly, Cas12m effectors exhibit PAM diversity that includes both T- and C-rich sequences. So far, nuclease-deficient DNA binding activity of other CRISPR-Cas type V family effectors has only been reported to Cas12k effectors involved in Tn7-like transposon homing and several Cas12c variants (9,27,58). Here we show that DNA-binding activity without cleavage is a common feature of the Cas12m effectors, thereby expanding our understanding of the functionally diverse type V Cas12 protein family. Biochemical characterization of Cas12m orthologs has indicated their involvement in pre-crRNA maturation. However, the molecular details of this process remain unknown, as the D274A mutation in the MsCas12m RuvC active site did not abolish pre-RNA cleavage, and structural analysis of GoCas12m did not reveal an alternative active site that could be involved in pre-crRNA processing. Therefore, further studies are required to elucidate the mechanism of pre-crRNA maturation in V-M CRISPR-Cas systems.

Typically, nucleic acid interference provided by type V CRISPR-Cas systems relies on the cleavage of invading DNA. Cleavage-incompetent MmCas12m and Cas12c have been reported to utilize binding-induced DNA interference against MGEs (29,58). Our results indicate that Cas12m effectors can efficiently protect *E. coli* from plasmid and virus propagation by blocking MGE replication or transcription of essential genes (Figure 2A–D). The observed DNA interference mechanism provided by CRISPR-Cas12m could be advantageous for silencing MGEs or integrated prophages to reduce the fitness costs associated with nuclease activity in cells.

Interestingly, the GoCas12m containing the H-D-D variant of the RuvC active site showed more robust inhibition of plasmid and bacteriophage propagation and transcriptional repression of the sfGFP reporter compared to the catalytically-dead dAsCas12a variant, implying enhanced target-binding activity of Cas12m (Figure 2A–D). Detailed structural analysis of the GoCas12m-crRNA-target DNA complex revealed the typical bi-lobed architecture of the GoCas12m complex accommodating the DNA substrate, which has also been observed for other Cas12 and TnpB effectors (63,64,69–73). However, the GoCas12m protein exhibits a unique arginine-rich ‘Roof’ structural element in the REC and a ɑ-helical insertion of the RuvC domain for the interaction with the target DNA, suggesting that additional structural features besides RuvC inactivation have been acquired to support the binding-based DNA silencing function. The recently solved cryo-EM structure of the MmCas12m complex with bound target DNA shows similar structural elements suggesting a conserved DNA-binding mechanism for silencing adopted by Cas12m effectors (28). In addition, the ‘Roof’ motif also provides additional contacts to the gRNA-target DNA heteroduplex, extending up to 18 bp from the PAM sequence. In contrast, the closely related TnpB protein contacts 12 bp of the heteroduplex, suggesting that the observed increase in Rec lobe size by the ‘Roof’ insertion in Cas12m may represent the initial steps in the evolution of Cas12 effectors from TnpB to perform adaptive immunity function.

Finally, by taking advantage of the unique molecular features of Cas12m proteins, we have successfully repurposed GoCas12m by fusing it with adenine deaminase for targeted base editing in both *E. coli* and human cells. Importantly, GoABE (845 aa) showed up to 20% A-to-G editing at endogenous targets in the human genome comparable to the efficiency of a significantly larger enAsABE (1545 aa) variant (68). The sequencing revealed the preferred PAM-proximal editing window of GoABE, which is distinct from the PAM-distal A-to-G editing of enAsABE. This finding is consistent with the cryo-EM structure of GoCas12m ternary complex, where only the 2nd–5th PAM-proximal NTS bases are exposed to the solvent, whereas the PAM-distal NTS bases are shielded by the ‘Roof’ insertion (Figure 3B). Overall, the engineered GoABE variant offers the expanded targeting space of A-to-G editing within the current ABE toolbox.

Collectively, these results reveal that DNA-binding Cas12m effectors are active in *E. coli* and human cells and can be adopted as a platform for the development of compact base editors compatible with a single AAV-vector delivery.

## Supplementary Material

gkae016_Supplemental_Files

## Data Availability

The electron density maps have been deposited to the Electron Microscopy Data Bank under the accession number EMD-17757. The atomic coordinates and structural data have been deposited to the Protein Data Bank under the accession number 8PM4. Sequencing data have been deposited on the NCBI Sequence Read Archive under BioProject ID PRJNA1017386. Scripts for data analysis and visualization have been deposited at Zenodo (39).

## Notes

Present address: Rimante Zedaveinyte, Department of Biochemistry and Molecular Biophysics, Columbia University, New York, NY 10032, USA.
