# Supplementary material for: Innate programmable DNA binding by CRISPR-Cas12m effectors enable efficient base editing
Source: Nucleic Acids Res. 2024 Jan 23;52(6):3234–48. doi: 10.1093/nar/gkae016 (PMC11013384; doi:10.1093/nar/gkae016)
Supplement: gkae016_Supplemental_Files [file gkae016_supplemental_files.zip › 2023_Bigelyte_et_al_SI_updated.pdf]

SUPPLEMENTARY INFORMATION

Supplementary Information includes 12 figures and 10 tables.

Figures:

- Figure S1. Analysis of Cas12m bound RNAs.
- Figure S2. pre-crRNA cleavage by Cas12m.
- Figure S3. PAM plasmid library cleavage assay by Cas12m.
- Figure S4. Cas12m DNA binding and R-loop formation.
- Figure S5. Cas12m nuclease activity testing.
- Figure S6. CRISPR-Cas12m activity in *E. coli* cells.
- Figure S7. Cryo-EM single particle reconstruction of the GoCas12m-crRNA-DNA ternary complex.
- Figure S8. Structural features and DNA target recognition by GoCas12m.
- Figure S9. Structural comparison of TnpB, Cas12m, and Cas12a ternary complexes.
- Figure S10. Structural comparison of GoCas12m and MmCas12m ternary complexes.
- Figure S11. Flow cytometry analysis of GoABE and enAsABE activity in human cells.
- Figure S12. GoABE and enAsABE base editing activity in human cells.

Tables:

- Table S1. Cas12m family representatives used in this study (additional file “Supplementary Table S1.xlsx”).
- Table S2. Cas12m proteins used in this study.
- Table S3. Sequences of proteins used in this study.
- Table S4. Plasmids used in this study.
- Table S5. RNAs used in this study.
- Table S6. DNA oligonucleotides used in this study.
- Table S7. Primers used in this study.
- Table S8. Target sequences used in this study.
- Table S9. Cryo-EM data collection, refinement and validation statistics for GoCas12m-crRNA-DNA ternary complex.
- Table S10. Flow cytometry results.

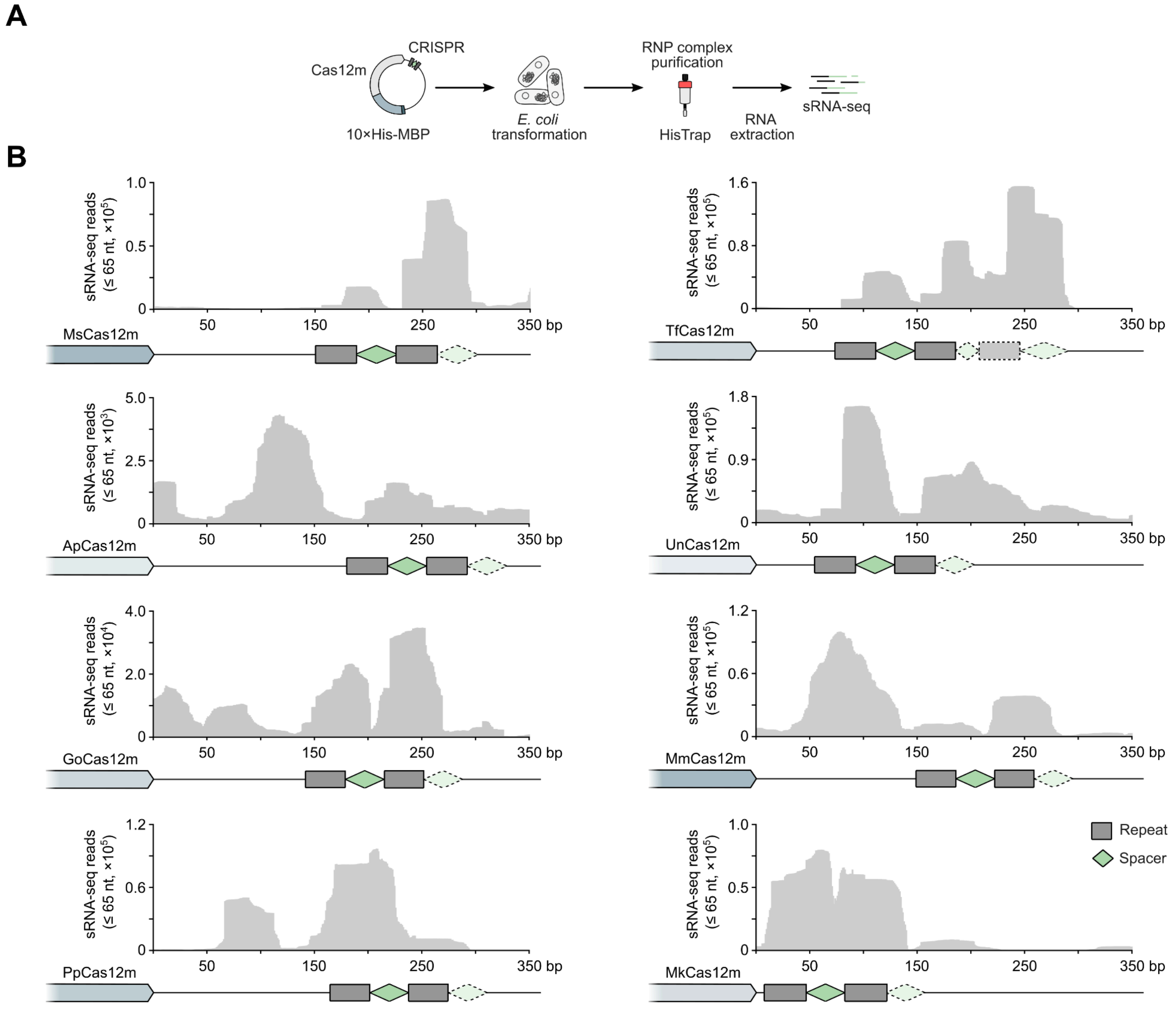

Figure S1. Analysis of Cas12m bound RNAs.

(A) Experimental workflow of the purification of the Cas12m RNP complexes from *E. coli* cells and extraction of bound RNAs.

(B) Mapping of sequenced small RNA (sRNA) reads to Cas12m CRISPR regions. The gray rectangle and green diamond represent repeat and spacer sequences, respectively.

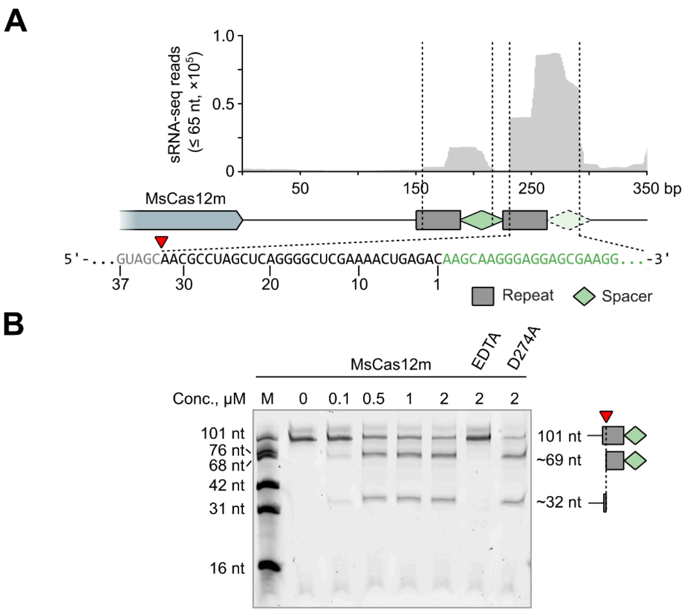

Figure S2. pre-crRNA cleavage by Cas12m.

(A) Mapping of sequenced sRNA reads to the MsCas12m CRISPR region.

(B) pre-crRNA cleavage by MsCas12m. D274A represents the MsCas12m variant with D274A mutation at the RuvC active site. The gray rectangle and green diamond represent repeat and spacer sequences, respectively. The red triangle indicates a cleavage site. M – RNA size marker.

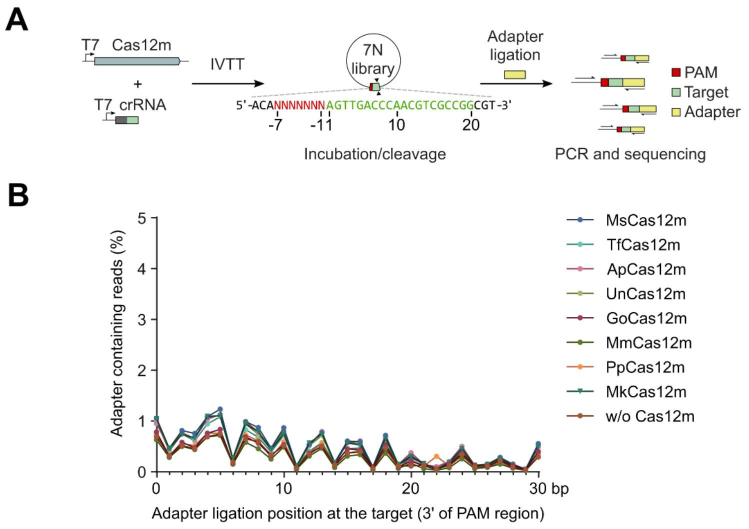

Figure S3. PAM plasmid library cleavage assay by Cas12m.
(A) Experimental workflow to detect dsDNA cleavage. Cas12m and crRNA designed to target the PAM library were synthesized using an *in vitro* transcription-translation (IVTT) system. The resulting solution containing Cas12m RNP complexes was used for the cleavage of the PAM library, followed by double-stranded break capture through adapter ligation, PCR amplification, and sequencing.
(B) Determination of adapter ligation positions in the targeted sequence. Compared to the negative controls (w/o Cas12m), none of the tested Cas12m RNP complexes designed to target a PAM library produced an enrichment in the adapter-ligated fragments.

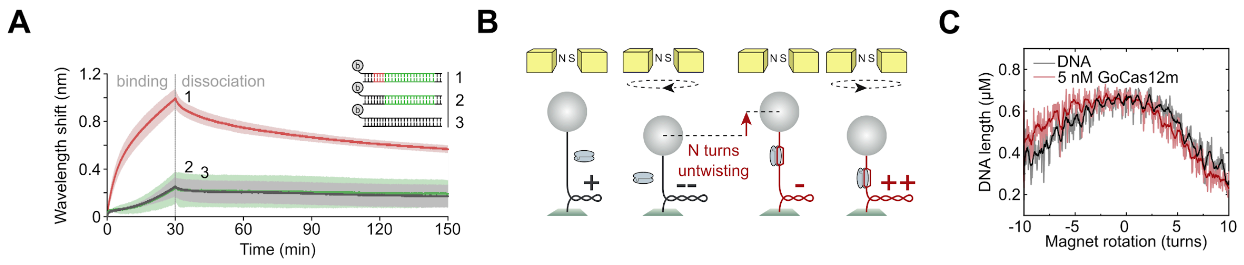

Figure S4. **Cas12m DNA binding and R-loop formation.**

(A) BLI sensorgrams showing GoCas12m association with and dissociation from dsDNA substrates. Representative sensorgrams of three independent measurements are displayed (mean ± SD).

(B) Schematic representation of the magnetic tweezers DNA twisting assay to detect R-loop formation by GoCas12m. Twisting single magnetic bead-tethered DNA molecules using a pair of magnets provides a significant DNA length reduction due to the formation of writhe. Upon DNA untwisting during R-loop formation negative supercoiling is absorbed providing a characteristic length change. To probe stable R-loop formation positive supercoiling can be applied subsequently.

(C) DNA length when twisting the DNA from negative to positive supercoiling at a force of 0.3 pN. The curve in the absence of enzyme (black) is centered around 0 turns while the curve in the presence of enzyme (red) is shifted by -1.9 turns demonstrating that GoCas12m successfully formed R-loops of ~20 bp. Notably, the shift of the supercoiling curve persisted also at positive supercoiling which indicates stable R-loop formation that can resist positive twist.

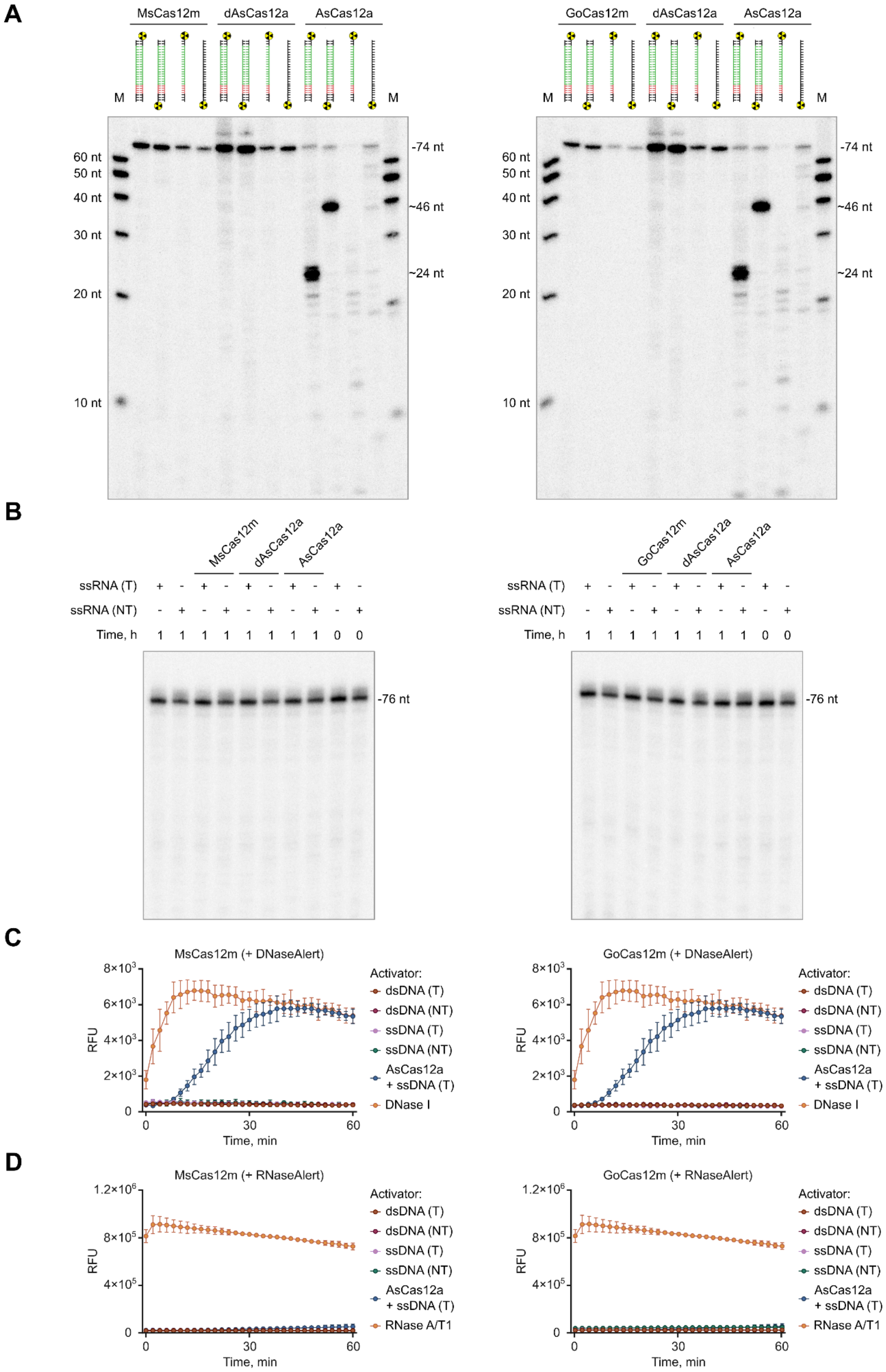

Figure S5. Cas12m nuclease activity testing.

(A) DNA cleavage assay by MsCas12m and GoCas12m. Target and PAM sequences are shown in green and red colors, respectively. dAsCas12a – AsCas12a RuvC active site mutant (D908A), M – radiolabeled DNA size marker.

(B) ssRNA cleavage assay by MsCas12m and GoCas12m. T and NT – ssRNA substrates with and without target sequences.

(C and D) Testing of MsCas12m and GoCas12m trans-cleavage activity of non-specific DNA (C) and RNA (D) substrates using dsDNA and ssDNA activators. Data are presented as mean ± SD (n = 3). T and NT – DNA activators with and without target-containing DNA sequences, respectively. RFU – relative fluorescence unit.

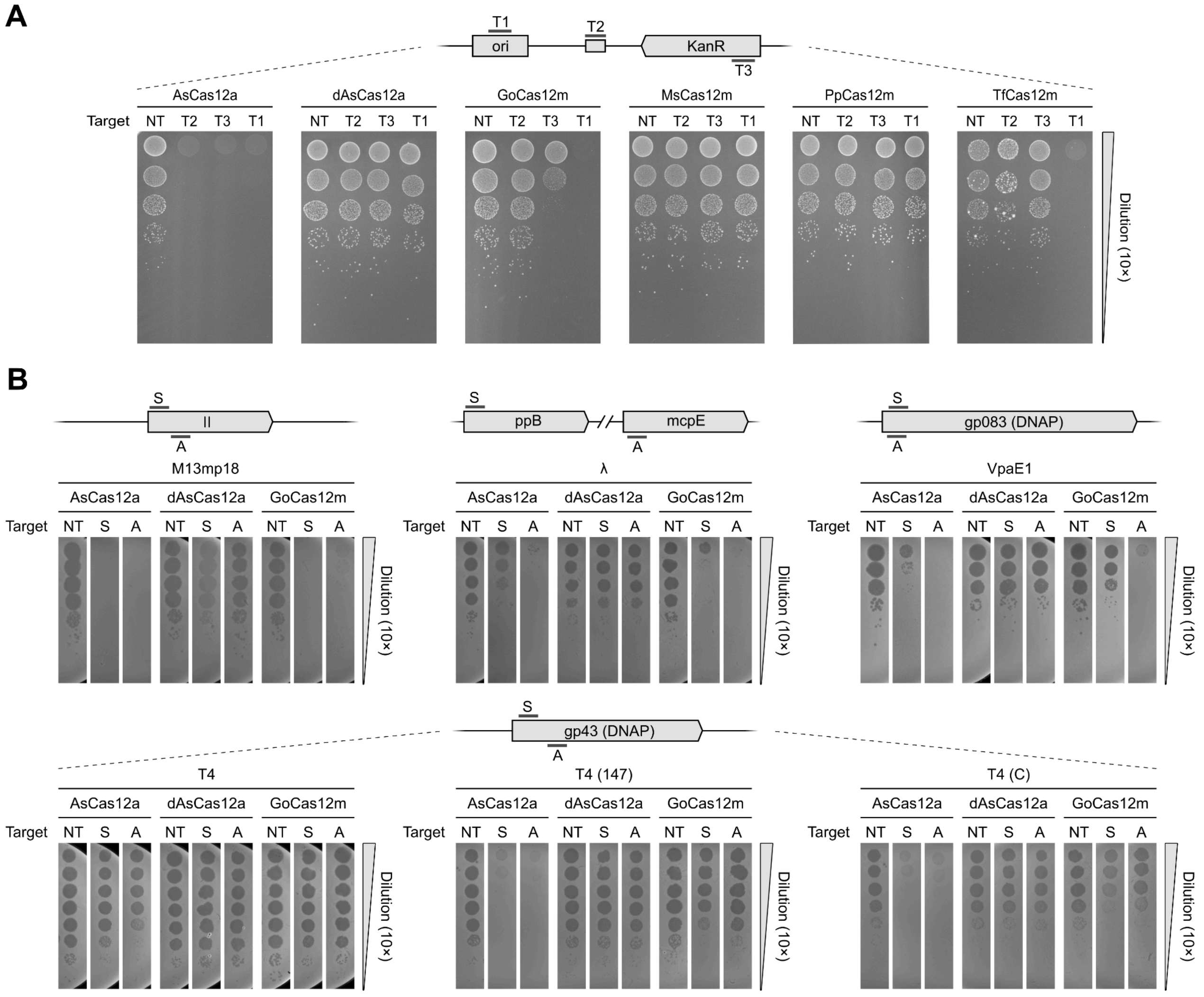

Figure S6. CRISPR-Cas12m activity in *E. coli* cells.

(A) Plasmid DNA interference assay in *E. coli*. To assess transformation efficiency, each *E. coli* transformant sample was serially diluted (10×) and grown on the Kn supplemented media at 37 °C overnight. KanR – kanamycin resistance, ori – origin of replication, dAsCas12a – AsCas12a RuvC active site mutant (D908A), T and NT indicate targeting and non-targeting crRNA constructs, respectively.

(B) Bacteriophage plaque formation assay in *E. coli*. To assess the efficiency of plating (EOP), phages were serially diluted (10×) and spotted onto lawns of *E. coli* expressing AsCas12a, dAsCas12a or GoCas12m. Effective reduction of phage infection resulted in a reduction of plaque-forming units. S and A indicate targeted sense and antisense DNA strands, respectively.

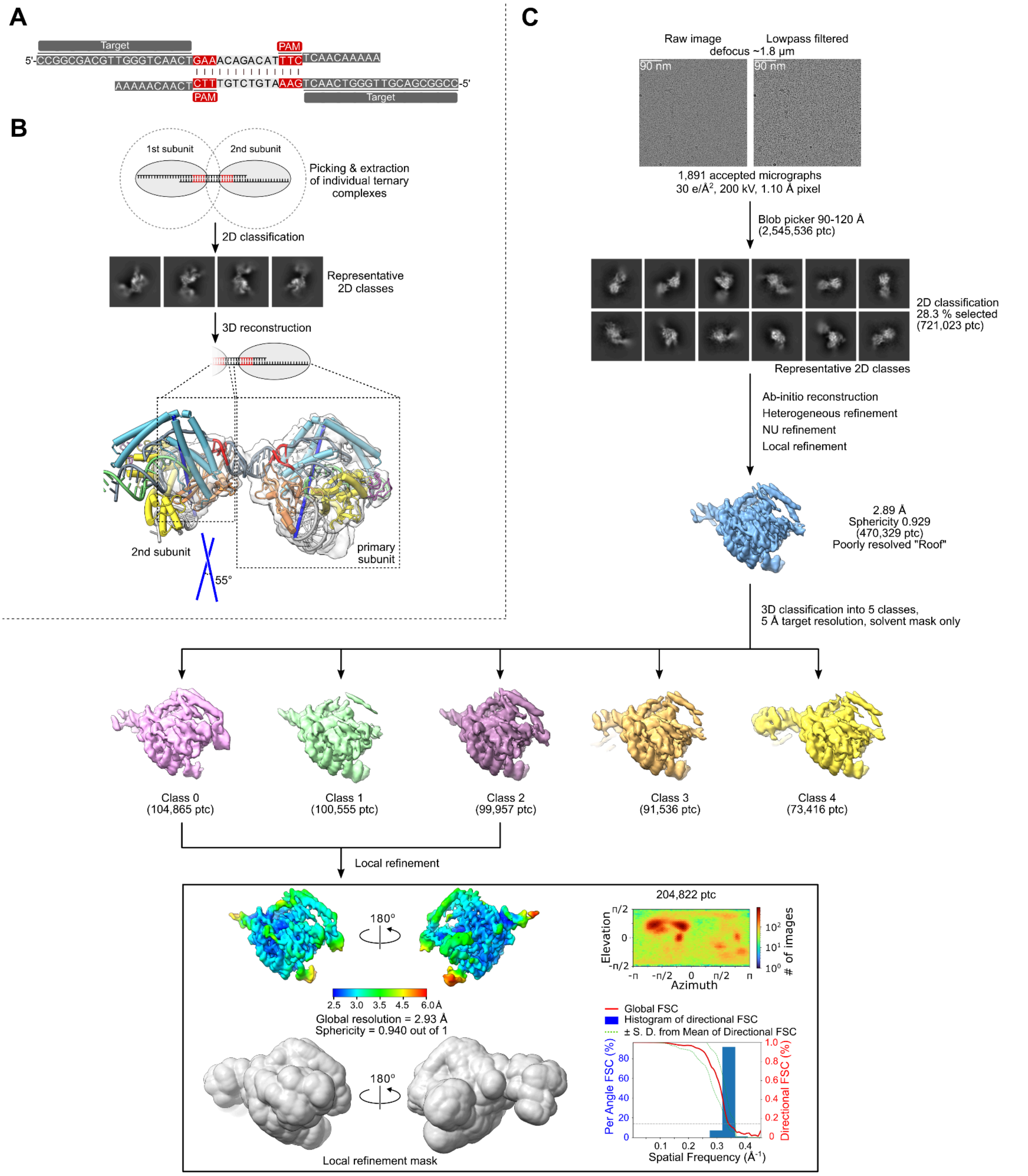

Figure S7. Cryo-EM single particle reconstruction of the GoCas12m-crRNA-DNA ternary complex.
(A) Design of the head-to-head dual-end DNA oligoduplex that was used for structural analysis of the GoCas12m-crRNA-DNA ternary complex.
(B) Interpretation of the cryo-EM data obtained with the dual-end DNA. During data processing, the GoCas12m RNP complexes formed at each terminus of the dual-end DNA were interpreted as individual particles as described previously (1). The sequences of the oligonucleotides are listed in Table S6.
(C) Workflow of the cryo-EM image processing and reconstruction for the GoCas12m-crRNA-DNA ternary complex. The final electron density maps showing local resolution, masks from the local refinement jobs, directional distribution plots and FSC (Fourier shell correlation) plots are shown in black rectangle.

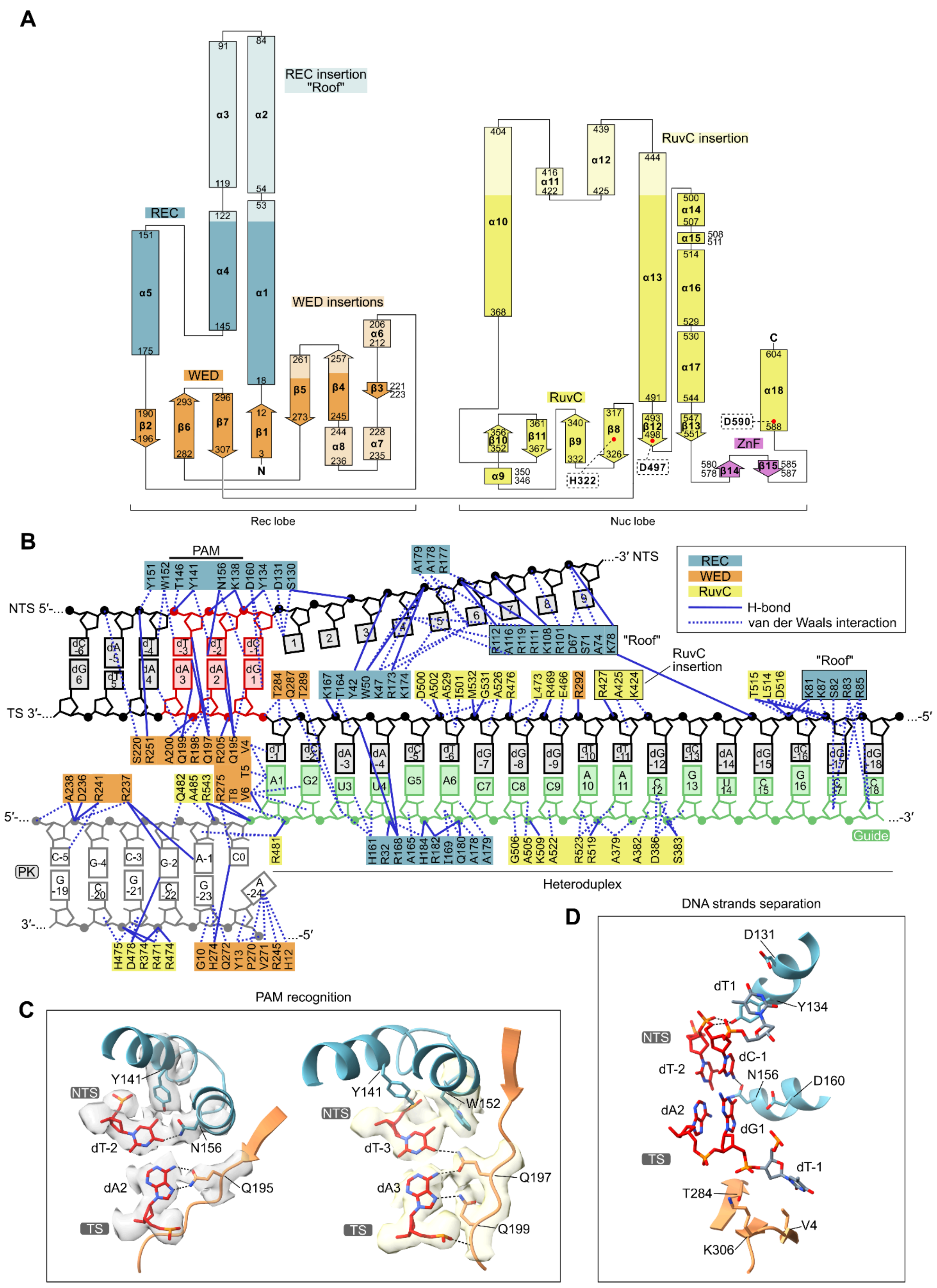

Figure S8. Structural features and DNA target recognition by GoCas12m.

(A) Topology diagram of the GoCas12m structure. Red dots indicate putative RuvC active site amino acids.

(B) Schematic representation of crRNA and DNA target recognition by GoCas12m.

(C) Structural details of 5’-TT-3’ sequence recognition in 5’-TTN-3’ PAM motif.

(D) The zoomed-in view of the DNA strands separation and phosphate inversion adjacent to the PAM sequence. TS – target strand, NTS – non-target strand.

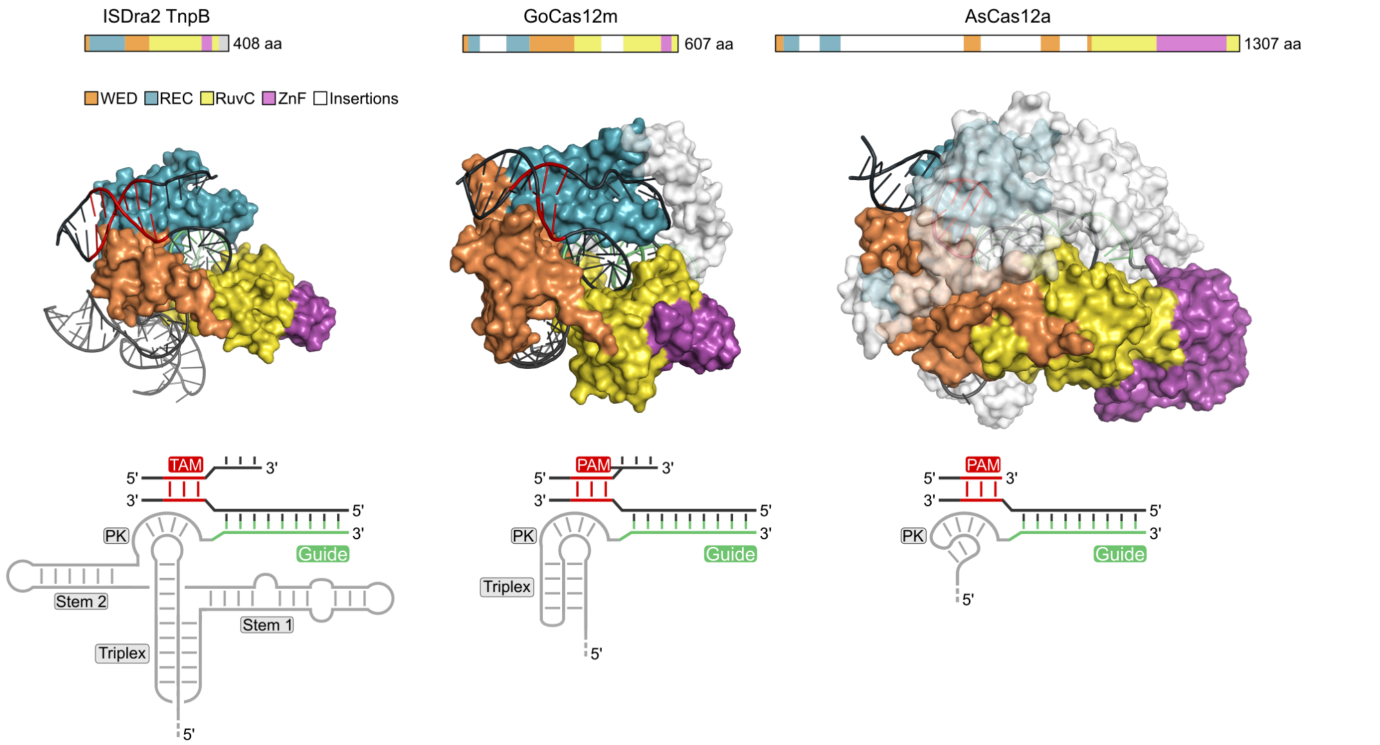

Figure S9. Structural comparison of TnpB, Cas12m, and Cas12a ternary complexes.
Comparison of domain organization (top), structures (middle), and guide RNAs (bottom) of ISDra2 TnpB (PDB ID: 8EXA), in this study obtained GoCas12m (PDB ID: 8PM4) and AsCas12a (PDB ID: 5B43). TAM – transposon adjacent motif, PAM – protospacer adjacent motif, and PK – pseudoknot.

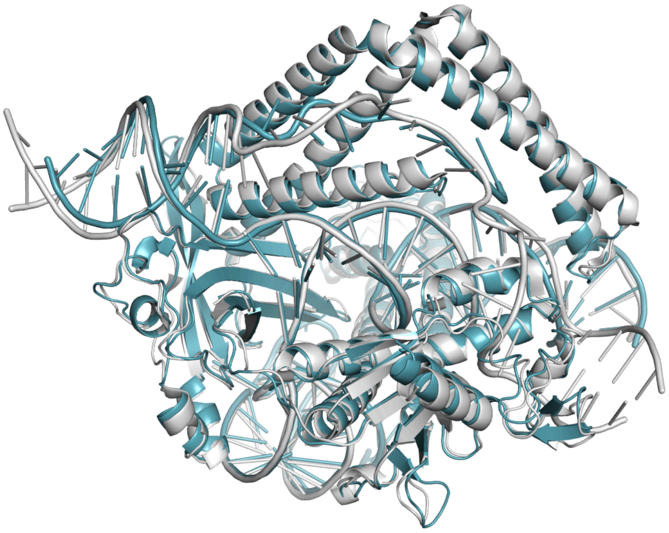

Figure S10. Structural comparison of GoCas12m and MmCas12m ternary complexes.
The superposition of GoCas12m (colored in light blue, PDB ID: 8PM4) with MmCas12m (colored in grey, PDB ID: 8HHL) structures shows a high structural similarity (RMSD of 1.1 Å for 577 equivalent Cα atoms).

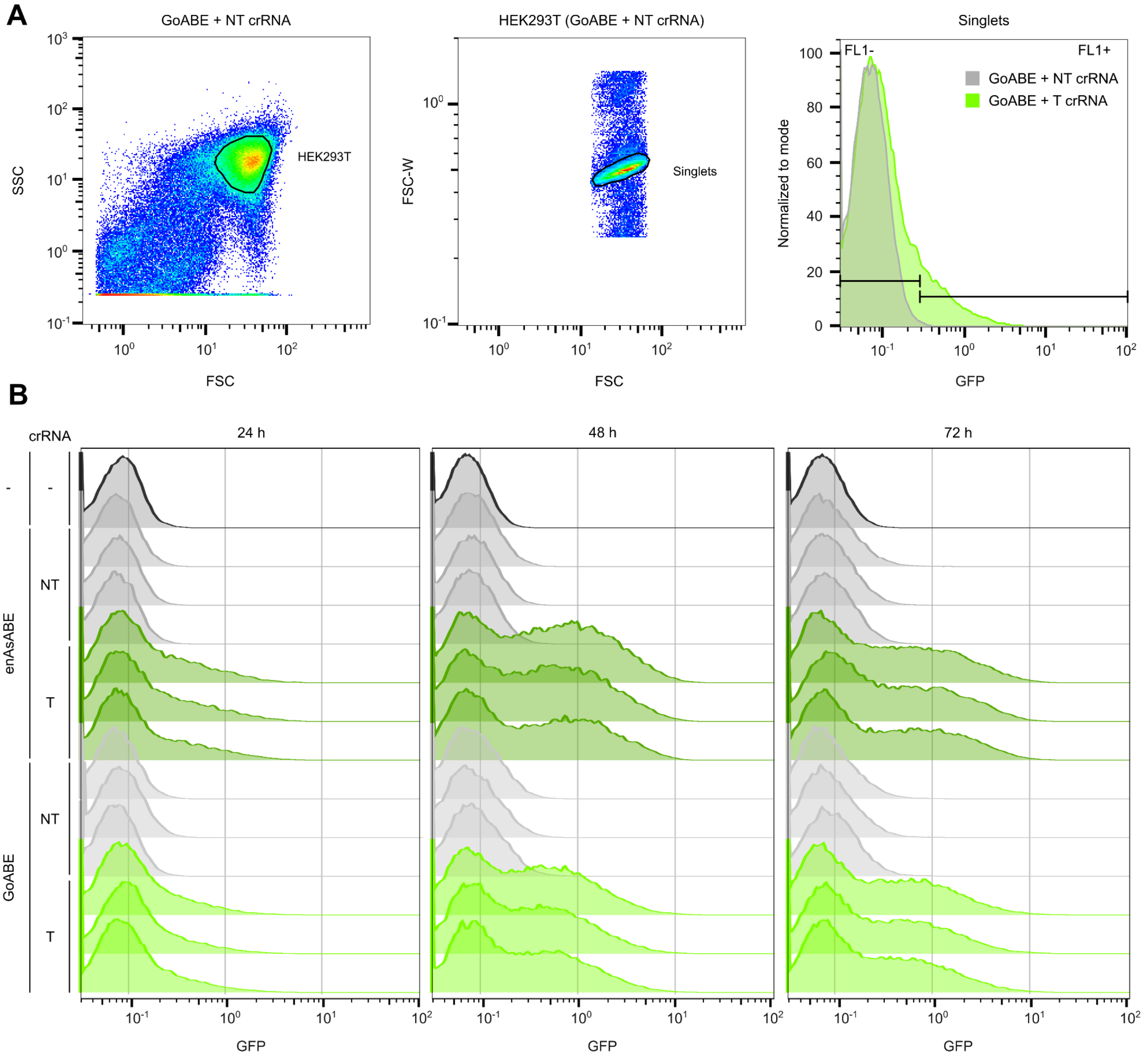

Figure S11. Flow cytometry analysis of GoABE and enAsABE activity in human cells.
(A) Gating strategy. Data of gated HEK293T cell singlets were plotted in histograms with at least 20,000 singlets analysed. The percentage of GFP-positive cells was determined by comparing paired sample groups of cells transfected with either targeting (T) or non-targeting (NT) crRNA constructs. SSC – side scatter, FSC – forward scatter, and FSC-W – FSC width.
(B) Flow cytometry analysis of GFP-positive HEK293T cells after 24, 48 and 72 h.

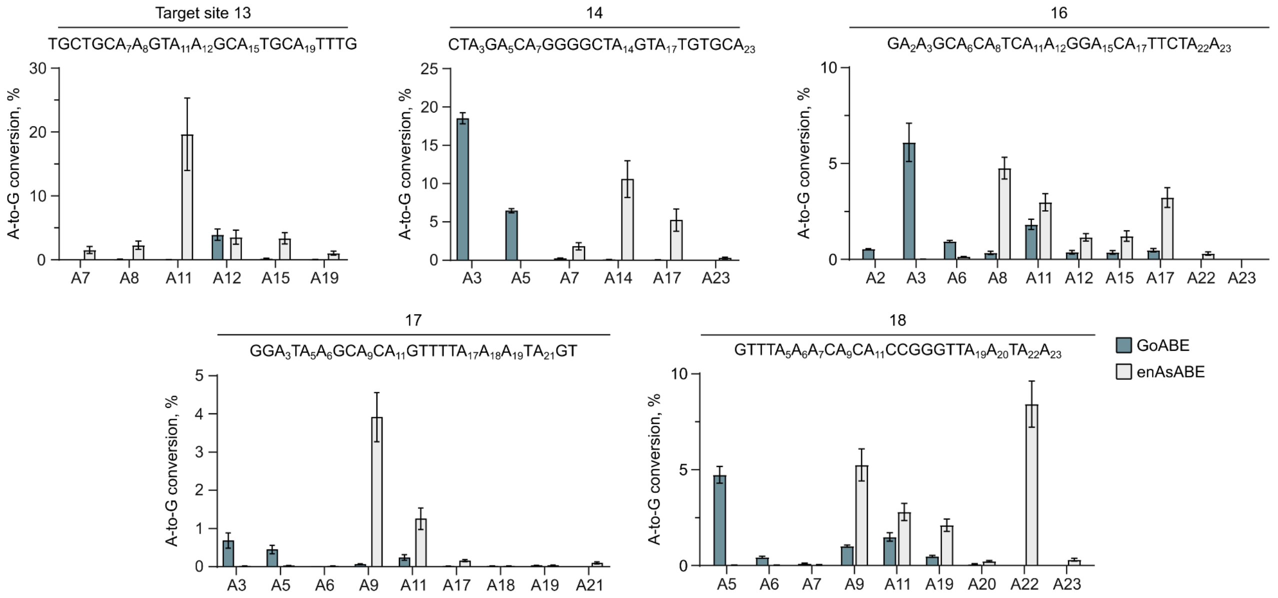

Figure S12. GoABE and enAsABE base editing activity in human cells.
DNA targets annotated according to Richter et al. 2020 (2). Data are presented as mean ± SD (n = 3).

Table S2. Cas12m proteins used in this study.

Name	RuvC	Size (aa)	Molecular mass (kDa)	Protein id (NCBI)	Organism	Scaffold accession (NCBI or IMG/VR)
GoCas12m	H322, D497, D590	607	67.6	GAB36148.1	*Gordonia otitidis* NBRC 100426	BAFB01000202.1
MmCas12m	H317, D485, D579	596	66.2	WP_061006603.1	*Mycolicibacterium mucogenicum*	NZ_LSKA01000495.1
MkCas12m	N292, D447, D539	589	66.8	WP_096876841.1	*Methylomonas koyamae* strain LM6	NZ_CP023670.1
MsCas12m	D274, E429, D518	536	61.5	WP_013159911.1	*Meiothermus silvanus* DSM 9946	NC_014214.1
ApCas12m	D353, E516, D602	628	71.2	TMJ00921.1	*Alphaproteobacteria*	VBAH01000059.1
UnCas12m	D304, E457, D548	583	67.2	Ga0104756_100012943	Uncharacterized IMGVR_Ga0104756_100012943	3300007352
PpCas12m	N408, D505, D594	606	68.6	WP_011733919.1	*Pelobacter propionicus* DSM 2379	NC_008607.1
TfCas12m	D306, E459, D550	567	66.5	QNB45423.1	*Thermoanaerosceptrum fracticalcis*	CP045798.1

Table S3. Sequences of proteins used in this study.

GoCas12m
MTRVTVQTAGVHYKWQMPDQLTQQLRLAHDLREDLVTLEYEYEDAVKAVWSSYPAVAALEAQVAELDERASELASTVKEEKSRQRTKRPSHPAVAQLAETRA<br>QLKAAKASRREAIASVRDEATERLRTISDERYAAQKQLYRDYCTDGLLYWATFNAVLDHHKTAVKRIAAHRKQGRAAQLRHHRWDGTGTISVQLQRQATDPART<br>PAIIADADTGKWRSSLIVPWVNPDVWDTMDRASRRKAGRVVIRMRCGSSRNPDGTKTSEWIDVPVQQHRMLPADADITAAQLTVRREGADLRATIGITAKIPDQ<br>GEVDEGPTIAVHLGWRSSDHGTVVATWRSTEPLDIPETLRGVITTQSAERTVGSIVVPHRIEQRVHHHATVASHRDLAVDSIRDTLVAWLTEHGPQPHPYDGDPI<br>TAASVQRWKAPRRFAWLALQWRDTPPPEGADIAETLEAWRRADKKLWLESEHGRGRALRHRTDLHRQVAAYFAGVAGRIVVDDSDIAQIAGTAKHSELLTDVD<br>RQIARRRAIAAPGMLRAAIVAAATRDEVPTTTVSHTGLSRVHAACGHENPADDRYLMQPVLCDGCGRTYDTDLSATILMLQRASAATSN*
MmCas12m
MTTMTVHTMGVHYKWQIPEVLRQQLWLAHNLREDLVSLQLAYDDDLKAIWSSYPDVAQAEDTMAAAEADAVALSERVKQARIEARSKKISTELTQQLRDAKKR<br>LKDARQARRDAIAVVKDDAAERRKARSDQLAADQKALYGQYCRDGDLYWASFNTVLDHHKTAVKRIAAQRASGKPATLRHHRFDGSGTIAVQLQRQAGAPPR<br>TPMVLADEAGKYRNVLHIPGWTDPDVWEQMTRSQCRQSGRVTVRMRCGSTDGQPQWIDLPVQVHRWLPADADITGAELVVTRVAGIYRAKLCVTARIGDTEP<br>VTSGPTVALHLGWRSTEEGTAVATWRSDAPLDIPFGLRTVMRVDAAGTSGIIVVPATIERRLTRTENIASSRSLALDALRDKVVGWLSDNDAPTYRDAPLEAATV<br>KQWKSPQRFASLAHAWKDNGTEISDILWAWFSLDRKQWAQQENGRRKALGHRDDLYRQIAAVISDQAGHVLVDDTSVAELSARAMERTELPTEVQQKIDRRR<br>DHAAPGGLRASVVAAMTRDGVPVTIVAAADFTRTHSRCGHVNPADDRYLSNPVRCDGCGAMYDQDRSFVTLMLRAATAPSNP*
MkCas12m
MIRTYKYSLKAPENFAEDCEDELRRMNDLWNRLIEIDRQRERSFKDLCRSTSAEYAAAQDEIEALREPIDNLYDAIRAERIATRSKEPSDELRARRDELLGRRKAL<br>WEICKAIQKAIPKESQAPINEVYKTNVKLARQQSGCFWGNYNAVIESFETAKSKAIKDGGRLHFKSFDGSGRFVNQIQGGMTVTELLAGSHSQAQLTNLVTTNK<br>TKGRFAFTAFTGKDDAGKRFRRQLFSEINYHRPIPADGVIKAVEVVKVPHDGKQKYKWHACFTVALPEVDIKHPKRNIAGVNLGWRQFGGRLRVAVVVDDAGK<br>KTEYFVPAELVSKFEAAETIQKAADDARNEMLSWLRTFYQDNRDEAPQEWRESIQGLLRNRPSVDAANHLMTIWRECVFAQEESRRYAAWLKSDAALRRSYT<br>GCRQNAVKWREEIYRHIAKELAERYAVLAVTDTPLSTMSRTKAKDDLAVDNALPESARRNRVIAAIYSLKEWIGKQAAKTGSTVETITGKMTATCHKCGYVAEKR<br>LRGSQYATCKSCGSELELDENAAINCRNHASGAVLISDKPEKTGRFQRAKMAENDFARKIGDNASPLVT*
MsCas12m
MPFGKKARHVKAYQFGADAPQEGMEAVLEQHRLRTDYYNALVEMELRQREERTALLANLAAESGLESPNQVYERLKAAGEKGIRKHPEYVAARERQKALYGH<br>PRLLELQSRQREERNALRRSFGAKGLYSSNYLDVERAFDKARQSPELRFRRYSPHEGRLAVLYTEGLPMREIGSDTRVQLPLPDPIIYRDRATRRKHQRVLMKF<br>RVRSVERQPLWITVPVYLHRELPDGVCREVSLHWHRVADRLRWTVSVVVEVEGPPVASPTGRGAVAVDLGWRRVEGGLRAGFWVGEDGAGGEIALSEGDLK<br>QFSKVEDLRSIRDQHLNALKEALAAWLEAPPAPLPDWLAEETKTLPQWRSPARFAALFRRWQSERVHADEAAYGLLEGWHKRDRHLWQYEANLREQMILRRR<br>EQYRVLAATLARQYDALIVEDFNLRAAAELDQGGSDLPDAARRYRTIASPSTLRDALVNAFAQRGKPVRKLNPAHTTTDCHACGGALVGDPAKELRLYCPTCER<br>FYDQDENAARNLLRRAQEVQAQV*
ApCas12m
MSEQLDDTPEQPNEVEETKKRKQRNKGKHPARIWSVFSRYLVSGREHFDKQVLLAHRFRNKLVELELQRRAAANVVIAQASSELQPLIDALAAAEQVLEVSLQ<br>ELKAVRAKHRRRAESAAQRDAVTNARTARNQASKALSKARKDAFASEAAQVGLWLAEEHHFQAVLAARHAFINDGLYWPTATDVQDRARAMRKGAPPVFRRF<br>GGAEQAGRIAVQIQKSTDKSQSEGGITFEEAFSCSHGFFRLEKKPGRDPLPEIADQPDYKSKRQQLLTYARAWLRVGSEGKGARAKPCWVVADVLLTRQAPKT<br>ARIVQVYLDHSVIGDRERWRLSLVLTNQEGWPKPNRASGCMVGIDLGWRLLDTGELRVAYACGADGQHHELRLPASLVKVWRRPDRIQQERDNLFNDVKARL<br>LEWLKGREDLPDWLKEQAEHLHLWKSSTRLSRLVDHWAGRDINWSSQRRIAGDEEILASLRGWVKRNLHLRDYQYHEREQLAAHRLDVYRKWADGLARLYQ<br>TAVLEDADWRDLARLPSPEDDAVNETARYNQRMASPGLLASVITNMFAITSRVECANTTRECWRCGHTEAFDAEAQLIRVCPGCGDACDQDESAARVLLARG<br>QALNQSQVAEAAPSS*

UnCas12m
MPFRKPAEPRDNRVYEYGCLPPTAGFDDMLDQLRGRNTFWNCLVEIERRQRIEVRSLLQPPESKVPALQQELEEARVAIKAERKRQRSRNADVSDLRAYIKDL<br>QQQLKSARAEDKAARERIKEASQPRLDELEAERLEQVKAAQHNSNLYWCNYDDVLASYGVARVRAMREGTEIKFHRFDGTGKVSVRYQQGLPVADVFGADT<br>RLQIRPVDPRAWTSPIRGERRRLARTVVRIRIGSHEDRSPVWLELPMVMHRPLPEGGGIRNAAVVRERIADSWRYRLVITVATPQQEIVTHQDRNRIAIDVGWRK<br>LDEGLRVAYWVDDLGQQGQVLLANEVIEQFRKVDDLKSIRDLHFNATRVALVDWLQHVVVPDWLAERCKTLSFWQSQARLSALVRDWEGQRFDADDEMLAG<br>LQAWWVKDRHLWQWESHLRDQVGRHRRELYRRFAADLVRRYRQVVLEDFDLRRVIEHPKAEVGTGGGIPGAWYRKTAAISSLRLTIEHTCSREGVEVLKVNP<br>SYSTLTCHVCGQIDQFNAAAELVHKCSQCGLLWDQDANAAAVLLQRASQIEAVSEQKTSCEESTTPPTDGG*
PpCas12m
MIAPTRTEKYLYLVPDEQVQPVTTIVRKYGLLSPLDWDCPDYPAGDAFEHLFLQNKLWNDLVTIEREHRAKYRELIGSDEETAQMDTEIASIKDRLSVLDEGRKKL<br>RVEHRKKKCPEIDCLDENIKKLKSELKAVASKAKETRAAAKDRIRAAGNDIENLEKDRQAAVIKAYNNSGLWWGNYNAVLESYKKARIKALKDGAELKYHRFDGS<br>GRFTNQIQGGMSVQDLLEGNRNVASLRLVSSGELGDISGKKPPSLDLQSVGSRRDSREYGILAITLYTGTDEQSKKFRRTLSFPVILHRPLPEGATLKSLSVHRK<br>RVGTDFVWSVVFTFTTDCPTYDQRSSTGNRCGLNLGWKKQAGGGLRVATIYDGSDARHITLPQAIIDGLDYVNGDLQGRIDSAANENHAWLLEQWGGDELPE<br>SLQELRSMLRRSKRPHPAKFAKAVIAWRNYPEYLGDARDEAEQRRKATKRLTIEMAHKREKLLRRRMDFYRNTAKQLTSVYDVICLDKMDLRRLALLEKGDGTP<br>NELTKIARKQRQQAISELRECLSKAAAKNGTQIEQVSTASSATCSACKGKMEQVDGIMWRCRECRALVDQDINAAANLFREVL*
TfCas12m
METAATKNYLALSFGCLSPTRGEEYLLDQIKKKHDLWNKLVEKDREHREKVRQVMVFESETTKKIKELEEELNSLREEIKNQRKTKRTGKVDLTDQKARIEEIKP<br>QLKQLKEKFKEERSFIFEARKQELAQLEKERWAVVKELGKGSGLYWCNLEDVVNSYDIGRKKAKAAGGEMRFHRWDGTGKVTVRFQKGLPVNEMFSCTNNL<br>LQIDPVDKDAWYNPVRAIRRKKSRTRVRLRACSENKKPLFIELPVVLHREIPEDALIRTASVIREKVGMRYRYKLNLVLEILGENTNRILPALEGTAAIDLGWRTVK<br>DGLRVACLVDDKGHSEELILDNDVLHEFNKIKDLQSIRDNLFNETKAKLMELLKTLELPDEAKERTSHMANWRSQQKMLRLHQYWRENRLPGDDEVWEVLEY<br>WRKREIHLYEWQENLRDQVLRRRKEIYRIFAAKITRKYKTIVLEEFTLNKTVQKPNPEEGPAGTLPANRNRFIAAISEFRNELANACRKNHVEFTYVPAENTTITCH<br>KCGHKEKFDAAAQIIHTCSTCGELWDQDYNAAKNLLAFSQKGGVK*
AsCas12a
MTQFEGFTNLYQVSKTLRFELIPQGKTLKHIQEQGFIEEDKARNDHYKELKPIIDRIYKTYADQCLQLVQLDWENLSAAIDSYRKEKTEETRNALIEEQATYRNAIH<br>DYFIGRTDNLTDAINKRHAEIYKGLFKAELFNGKVLKQLGTVTTTEHENALLRSFDKFTTYFSGFYENRKNVFSAEDISTAIPHRIVQDNFPKFKENCHIFTRLITAV<br>PSLREHFENVKKAIGIFVSTSIEEVFSFPFYNQLLTQTQIDLYNQLLGGISREAGTEKIKGLNEVLNLAIQKNDETAHIIASLPHRFIPLFKQILSDRNTLSFILEEFKSD<br>EEVIQSFCKYKTLLRNENVLETAEALFNELNSIDLTHIFISHKKLETISSALCDHWDTLRNALYERRISELTGKITKSAKEKVQRSLKHEDINLQEIISAAGKELSEAFK<br>QKTSEILSHAHAALDQPLPTTLKKQEEKEILKSQLDSLLGLYHLLDWFAVDESNEVDPEFSARLTGIKLEMEPSLSFYNKARNYATKKPYSVEKFKLNFQMPTLAS<br>GWDVNKEKNNGAILFVKNGLYYLGIMPKQKGRYKALSFEPTEKTSEGFDKMYYDYFPDAAKMIPKCSTQLKAVTAHFQTHTTPILLSNNFIEPLEITKEIYDLNNP<br>EKEPKKFQTAYAKKTGDQKGYREALCKWIDFTRDFLSKYTKTTSIDLSSLRPSSQYKDLGEYYAELNPLLYHISFQRIAEKEIMDAVETGKLYLFQIYNKDFAKGH<br>HGKPNLHTLYWTGLFSPENLAKTSIKLNGQAELFYRPKSRMKRMAHRLGEKMLNKKLKDQKTPIPDTLYQELYDYVNHRLSHDLSDEARALLPNVITKEVSHEII<br>KDRRFTSDKFFFHVPITLNYQAANSPSKFNQRVNAYLKEHPETPIIGIDRGERNLIYITVIDSTGKILEQRSLNTIQQFDYQKKLDNREKERVAARQAWSVVGTIKD<br>LKQGYLSQVIHEIVDLMIHYQAVVVLENLNFGFKSKRTGIAEKAVYQQFEKMLIDKLNCLVLKDYPAEKVGGVLNPYQLTDQFTSFAKMGTQSGFLFYVPAPYTS<br>KIDPLTGFVDPFVWKTIKNHESRKHFLEGFDFLHYDVKTGDFILHFKMNRNLSFQRGLPGFMPAWDIVFEKNETQFDAKGTPFIAGKRIVPVIENHRFTGRYRDL<br>YPANELIALLEEKGIVFRDGSNILPKLLENDDSHAIDTMVALIRSVLQMRNSNAATGEDYINSPVRDLNGVCFDSRFQNPEWPMDADANGAYHIALKGQLLLNHL<br>KESKDLKLQNGISNQDWLAYIQELRN*
Twin-Strep-10×His-MBP-TEV tag used for Cas12m purification
MGGSAWSHPQFEKGGGSGGGSGGSAWSHPQFEKGSMGGSHHHHHHHHHHGMASMKIEEGKLVIWINGDKGYNGLAEVGKKFEKDTGIKVTVEHPDKLEE<br>KFPQVAATGDGPDIIFWAHDRFGGYAQSGLLAEITPDKAFQDKLYPFTWDAVRYNGKLIAYPIAVEALSLIYNKDLLPNPPKTWEEIPALDKELKAKGKSALMFNL

QEPYFTWPLIAADGGYAFKYENGKYDIKDVGVDNAGAKAGLTFLVDLIKNKHMNADTDYSIAEAAFNKGETAMTINGPWAWSNIDTSKVNYGVTVLPTFKGQPSKPFVGVLSAGINAASPNKELAKEFLENYLLTDEGLEAVNKDKPLGAVALKSYEEELAKDPRIAATMENAQKGEIMPNIPQMSAFWYAVRTAVINAASGRQTVDEALKDAQTNSSSNNNNNNNNNNLGIEENLYFQSNAGGGG
10×His-MBP-TEV tag used for Cas12a purification
HHHHHHHHHHGSSMKIEEGKLVIWINGDKGYNGLAEVGKKFEKDTGIKVTVEHPDKLEEKFPQVAATGDGPDIIFWAHDRFGGYAQSGLLAEITPDKAFQDKLYPFTWDAVRYNGKLIAYPIAVEALSLIYNKDLLPNPPKTWEEIPALDKELKAKGKSALMFNLQEPYFTWPLIAADGGYAFKYENGKYDIKDVGVDNAGAKAGLTFLVDLIKNKHMNADTDYSIAEAAFNKGETAMTINGPWAWSNIDTSKVNYGVTVLPTFKGQPSKPFVGVLSAGINAASPNKELAKEFLENYLLTDEGLEAVNKDKPLGAVALKSYEEELAKDPRIAATMENAQKGEIMPNIPQMSAFWYAVRTAVINAASGRQTVDEALKDAQTNSSSNNNNNNNNNNLGIEENLYFQSNA
bpNLS-TadA-8e-enAsCas12a-bpNLS
MKRTADGSEFESPKKKRKVSEVEFSHEYWMRHALTLAKRARDEREVPVGAVLVLNNRVIGEGWNRAIGLHDPTAHAEIMALRQGGLVMQNYRLIDATLYVTFEPCVMCAGAMIHSRIGRVVFGVRNSKRGAAGSLMNVLNYPGMNHRVEITEGILADECAALLCDFYRMPRQVFNAQKKAQSSINSGGSSGGSSGSETPGTSESATPESSGGSSGGSMTQFEGFTNLYQVSKTLRFELIPQGKTLKHIQEQGFIEEDKARNDHYKELKPIIDRIYKTYADQCLQLVQLDWENLSAAIDSYRKEKTEETRNALIEEQATYRNAIHDYFIGRTDNLTDAINKRHAEIYKGLFKAELFNGKVLKQLGTVTTTEHENALLRSFDKFTTYFSGFYRNRKNVFSAEDISTAIPHRIVQDNFPKFKENCHIFTRLITAVPSLREHFENVKKAIGIFVSTSIEEVFSFPFYNQLLTQTQIDLYNQLLGGISREAGTEKIKGLNEVLNLAIQKNDETAHIIASLPHRFIPLFKQILSDRNTLSFILEEFKSDEEVIQSFCKYKTLLRNENVLETAEALFNELNSIDLTHIFISHKKLETISSALCDHWDTLRNALYERRISELTGKITKSAKEKVQRSLKHEDINLQEIISAAGKELSEAFKQKTSEILSHAHAALDQPLPTTLKKQEEKEILKSQLDSLLGLYHLLDWFAVDESNEVDPEFSARLTGIKLEMEPSLSFYNKARNYATKKPYSVEKFKLNFQMPTLARGWDVNREKNNGAILFVKNGLYYLGIMPKQKGRYKALSFEPTEKTSEGFDKMYYDYFPDAAKMIPKCSTQLKAVTAHFQTHTTPILLSNNFIEPLEITKEIYDLNNPEKEPKKFQTAYAKKTGDQKGYREALCKWIDFTRDFLSKYTKTTSIDLSSLRPSSQYKDLGEYYAELNPLLYHISFQRIAEKEIMDAVETGKLYLFQIYNKDFAKGHHGKPNLHTLYWTGLFSPENLAKTSIKLNGQAELFYRPKSRMKRMAHRLGEKMLNKKLKDQKTPIPDTLYQELYDYVNHRLSHDLSDEARALLPNVITKEVSHEIIKDRRFTSDKFFFHVPITLNYQAANSPSKFNQRVNAYLKEHPETPIIGIARGERNLIYITVIDSTGKILEQRSLNTIQQFDYQKKLDNREKERVAARQAWSVVGTIKDLKQGYLSQVIHEIVDLMIHYQAVVVLENLNFGFKSKRTGIAEKAVYQQFEKMLIDKLNCLVLKDYPAEKVGGVLNPYQLTDQFTSFAKMGTQSGFLFYVPAPYTSKIDPLTGFVDPFVWKTIKNHESRKHFLEGFDFLHYDVKTGDFILHFKMNRNLSFQRGLPGFMPAWDIVFEKNETQFDAKGTPFIAGKRIVPVIENHRFTGRYRDLYPANELIALLEEKGIVFRDGSNILPKLLENDDSHAIDTMVALIRSVLQMRNSNAATGEDYINSPVRDLNGVCFDSRFQNPEWPMDADANGAYHIALKGQLLLNHLKESKDLKLQNGISNQDWLAYIQELRNSGGSKRTADGSEFEPKKKRKV*
bpNLS-TadA-8e-GoCas12m-bpNLS
MKRTADGSEFESPKKKRKVSEVEFSHEYWMRHALTLAKRARDEREVPVGAVLVLNNRVIGEGWNRAIGLHDPTAHAEIMALRQGGLVMQNYRLIDATLYVTFEPCVMCAGAMIHSRIGRVVFGVRNSKRGAAGSLMNVLNYPGMNHRVEITEGILADECAALLCDFYRMPRQVFNAQKKAQSSINSGGSSGGSSGSETPGTSESATPESSGGSSGGSMTRVTVQTAGVHYKWQMPDQLTQQLRLAHDLREDLVTLEYEYEDAVKAVWSSYPAVAALEAQVAELDERASELASTVKEEKSRQRTKRPSHPAVAQLAETRAQLKAAKASRREAIASVRDEATERLRTISDERYAAQKQLYRDYCTDGLLYWATFNAVLDHHKTAVKRIAAHRKQGRAAQLRHHRWDGTGTISVQLQRQATDPARTPAIIADADTGKWRSSLIVPWVNPDVWDTMDRASRRKAGRVVIRMRCGSSRNPDGTKTSEWIDVPVQQHRMLPADADITAAQLTVRREGADLRATIGITAKIPDQGEVDEGPTIAVHLGWRSSDHGTVVATWRSTEPLDIPETLRGVITTQSAERTVGSIVVPHRIEQRVHHHATVASHRDLAVDSIRDTLVAWLTEHGPQPHPYDGDPITAASVQRWKAPRRFAWLALQWRDTPPPEGADIAETLEAWRRADKKLWLESEHGRGRALRHRTDLHRQVAAYFAGVAGRIVVDDSDIAQIAGTAKHSELLTDVDRQIARRRAIAAPGMLRAAIVAAATRDEVPTTTVSHTGLSRVHAACGHENPADDRYLMQPVLCDGCGRTYDTDLSATILMLQRASAATSNSGGSKRTADGSEFEPKKKRKV*

Table S4. Plasmids used in this study.

Plasmid name	Description	Figures
Expression and purification of Cas12 proteins and RNP complexes		
pTK205	GoCas12m and minimal CRISPR region (https://benchling.com/s/seq-lVMwqGg4MvW22hAwWcAF?m=slm-feEdkhVjv53Zsze5M9Ub)	S1
pTK206	MmCas12m and minimal CRISPR region (https://benchling.com/s/seq-kxjaCNTfskQ08Lt5V7bZ?m=slm-lF4MvL07jaQVLA5pUx9v)	
pTK207	MkCas12m and minimal CRISPR region (https://benchling.com/s/seq-cHdyTlv5TSVOGCulWXWb?m=slm-bIU5IDpadNovErAylBeI)	
pTK208	MsCas12m and minimal CRISPR region (https://benchling.com/s/seq-s0Jhlvc433M8OYeDbuV2?m=slm-T8RQT049O9Wv5uaAnjpp)	S1; S2A
pTK213	ApCas12m and minimal CRISPR region (https://benchling.com/s/seq-VsJpOyrntglnhIqDZL6Y?m=slm-TXU4sRsnQPMuQU1ayofm)	S1
pTK214	UnCas12m and minimal CRISPR region (https://benchling.com/s/seq-iAeAKLVQ9q9yQUixqKSp?m=slm-Y1871mKkJWN123J6ZtiS)	
pTK218	PpCas12m and minimal CRISPR region (https://benchling.com/s/seq-UvnW4PZBylSVbeoaaase?m=slm-UtpqCNzLTVTATiltx3Rz)	
pTK219	TfCas12m and minimal CRISPR region (https://benchling.com/s/seq-DJWp0PwaYnPTjqhL6jcE?m=slm-XfNRdnZAj8PJdmQMJOPw)	
pMBP-AsCas12a	10×His-MBP-AsCas12a (https://www.addgene.org/113430/)	S5
pGB060	10×His-MBP-AsCas12a (D908A) (https://benchling.com/s/seq-KyViUDxiqZ0ycq1FUq9y?m=slm-6y3np62JraeiqEf57Etx)	
pTK200	Twin-Strep-10×His-MBP-GoCas12m (https://benchling.com/s/seq-DO9AF0YNKqbj7ilAkEdS?m=slm-3QqU1bqlpZUXH8KfVqJ1)	3; S4; S5; S7; S8; S9
pTK203	Twin-Strep-10×His-MBP-MsCas12m (https://benchling.com/s/seq-Tjium3suTdCRRWcpy4Uw?m=slm-PXygTnNqwF99s8PjpcOb)	S2B; S5
pTK201	Twin-Strep-10×His-MBP-MmCas12m (https://benchling.com/s/seq-448yhx9DgaSMPX5OALBa?m=slm-YaaJqEXB3bdBY8M1kcUK)	-
pTK202	Twin-Strep-10×His-MBP-MkCas12m (https://benchling.com/s/seq-DP9x96w0MN8iHBXYBEI4?m=slm-7A1ZjieuFBfUrmG9g0O1)	
pTK210	Twin-Strep-10×His-MBP-ApCas12m (https://benchling.com/s/seq-pQdvGTObkj3J542fwj9C?m=slm-wYtaTVsqLkeeXoHABNyl)	
pTK211	Twin-Strep-10×His-MBP-UnCas12m (https://benchling.com/s/seq-w5heCl4CBFrkD5sbvB8A?m=slm-n09uFbDqbMrZ5lFn1BW2)	
PAM identification assay		
pTZ57	7N PAM plasmid library (https://benchling.com/s/seq-rdFXhQRjKJZvBUuDZsjo?m=slm-Q8HwrqATauntmoywQmnM)	1D; S3

Cas12 expression in E. coli		
pTK147	Twin-Strep-10×His-MBP-AsCas12a (https://benchling.com/s/seq-F0GMxxiYFBpHY2RuGUnw?m=slm-Y8A8XHnmGNxss2EnQ9f4)	2; S6
pRZ169	Twin-Strep-10×His-MBP-AsCas12a (D908A) (https://benchling.com/s/seq-DrVN6Iv7hOF2oeZZw1BV?m=slm-m0KHMFWwiTcrysHQ73f9)	
pTK200	Twin-Strep-10×His-MBP-GoCas12m (https://benchling.com/s/seq-DO9AF0YNKgbj7iIAkEdS?m=slm-3QqU1bgIpZUXH8KfVqJ1)	
pTK203	Twin-Strep-10×His-MBP-MsCas12m (https://benchling.com/s/seq-Tjium3suTdCRRWcpy4Uw?m=slm-PXygTnNqwF99s8PjpcOb)	2A; S6A
pTK216	Twin-Strep-10×His-MBP-PpCas12m (https://benchling.com/s/seq-Vj18uTfenZI2KIrf7c61?m=slm-EJ7HbsyPESiOPUEFmT0A)	
pTK217	Twin-Strep-10×His-MBP-TfCas12m (https://benchling.com/s/seq-YT2f9W5TOaBqevUEcpUb?m=slm-aP6GIoTBeCufj0Vq5dxC)	
Plasmid interference assay in E. coli		
pKP008	AsCas12a and GoCas12m target plasmid (https://benchling.com/s/seq-ZxdyaoTCO3ZTHuQI1nxG?m=slm-aXMAkX8oGtKJ10X59w05)	2A; S6A
pKP009	MsCas12m target plasmid (https://benchling.com/s/seq-JWJn4s1WNf4xBAp6p4OB?m=slm-BSQMpBs5SBH0Cubz0Cxj)	
pKP011	PpCas12m and TfCas12m target plasmid (https://benchling.com/s/seq-UrxVpjK60T1eeW5v86gg?m=slm-xU6cUIVLXRNJKbvw5W43)	
pRZ166	AsCas12a crRNA (non-targeting) (https://benchling.com/s/seq-yaKOvcb1zfp9MuRHwZ82?m=slm-brib6CFbBDQbPBpOsPoo)	
pRZ167	GoCas12m crRNA (non-targeting) (https://benchling.com/s/seq-TBIB3mK3QHnUdpL3FkO6?m=slm-sEuUhkRqS6PkfHy0aP80)	
pRZ168	TfCas12m crRNA (non-targeting) (https://benchling.com/s/seq-4Yp97PodqX9Ay2DN0mP9?m=slm-tQ6ocpBW2Jg6v7mjICab)	
pRZ170	AsCas12a crRNA (T2 target) (https://benchling.com/s/seq-8Pk0vHDPzLRPuAKHdJqP?m=slm-6RWYFZIPoCIBAG5NkGII)	
pRZ171	AsCas12a crRNA (KanR T3 target) (https://benchling.com/s/seq-DQKeTCS608hdNXUda6KJ?m=slm-vTFDGfqo9pDgAWfuIMZX)	
pRZ172	AsCas12a crRNA (ori T1 target) (https://benchling.com/s/seq-Tr04LMAHW5hSzZ5XjxAc?m=slm-3y47M2Fg28OTD6UBPLmj)	
pRZ173	GoCas12m crRNA (T2 target) (https://benchling.com/s/seq-nbUKRMuvceYYmgouJbJE?m=slm-i4BSICTxeZw2FUOdjvq0)	
pRZ184	GoCas12m crRNA (KanR T3 target) (https://benchling.com/s/seq-qZdCVSwwISXYpIdBs4jU?m=slm-uiAL67mBh394qMqm95FC)	
pRZ185	GoCas12m crRNA (ori T1 target) (https://benchling.com/s/seq-pV2fJbiMoVmxUQtx5EwJ?m=slm-VqcFhsGrSUH8uICMPaAk)	

pRZ186	TfCas12m crRNA (T2 target) (https://benchling.com/s/seq-lHAtLd6NetEC7GzmkYrk?m=slm-hNGtMlawOr42M3eSH4i7)	
pRZ187	TfCas12m crRNA (KanR T3 target) (https://benchling.com/s/seq-P3c4DJsL3bIBAA9dVRTV?m=slm-bk0IUfC24ce82ZtI7ICj)	
pRZ188	TfCas12m crRNA (ori T1 target) (https://benchling.com/s/seq-n6D47JI6jird3F6IINK3?m=slm-CAIEImNBFf9LZeAfdEg0)	
pRZ194	MsCas12m crRNA (non-targeting) (https://benchling.com/s/seq-ykO416zY0OvkCwRgqcgF?m=slm-4uGp52YrZs0TFrDkp4nN)	
pRZ195	PpCas12m crRNA (non-targeting) (https://benchling.com/s/seq-FX4GBqNngOsdI4QVfPng?m=slm-FO0UXoi3wHUYBCJcWcEv)	
pRZ196	MsCas12m crRNA (T2 target) (https://benchling.com/s/seq-NwIvFRP3G0nbciGEVNqi?m=slm-TS5HcbiBKYahzMUZzUwf)	
pRZ197	MsCas12m crRNA (KanR T3 target) (https://benchling.com/s/seq-axJxoT8TFjv594uolwyy?m=slm-4COr8yq7AZrvgsi0I47R)	
pRZ198	MsCas12m crRNA (ori T1 target) (https://benchling.com/s/seq-HjZGS8lG6cEL7FCww6RK?m=slm-ziE08Y0XUpEEc01Kj0Ws)	
pRZ199	PpCas12m crRNA (T2 target) (https://benchling.com/s/seq-BnfvR5KssSHXtTIvhCOU?m=slm-gYqz85t2AsDy8VKjSZyP)	
pRZ200	PpCas12m crRNA (KanR T3 target) (https://benchling.com/s/seq-hXYomlMdZiUltx6HO3rW?m=slm-o1OiPntA6Dn7OjCqvtLj)	
pRZ201	PpCas12m crRNA (ori T1 target) (https://benchling.com/s/seq-uYr3LRCBYUcIQVUMdtvQ?m=slm-ngjiX4nm8OJeK3gr8p1K)	
	sfGFP silencing assay in E. coli	
pGB108	Target plasmid expressing sfGFP (https://benchling.com/s/seq-ekankPLhlipl933KtwKD?m=slm-LeHk0qzBoUWUHBA8309H)	
pRZ166	AsCas12a crRNA (non-targeting) (https://benchling.com/s/seq-yaKOvcb1zfp9MuRHwZ82?m=slm-brib6CFbBDQbPBpOsPoo)	
pRZ167	GoCas12m crRNA (non-targeting) (https://benchling.com/s/seq-TBlB3mK3QHnUdpL3FkO6?m=slm-sEuUhkRqS6PkfHy0aP80)	
pGB109	AsCas12a crRNA (sfGFP T1 target) (https://benchling.com/s/seq-eu1OAvkTbeAVfYPtKI7D?m=slm-qRXaGhKxVAPoCDr7jM1Y)	2C
pGB110	AsCas12a crRNA (sfGFP T2 target) (https://benchling.com/s/seq-WDDcvTMoHh4AMrDz6vl4?m=slm-krwcyolaygJ27Vxubu9k)	
pGB111	AsCas12a crRNA (sfGFP T3 target) (https://benchling.com/s/seq-cogSStJNSraeVbPlBBSX?m=slm-J9iEQjAnbYkdbm5lHPqK)	
pGB112	AsCas12a crRNA (sfGFP T4 target) (https://benchling.com/s/seq-YDtVUWmFq9iG4tjQ2g4S?m=slm-XYyl7SDmZboJqvPPHXZt)	
pGB113	AsCas12a crRNA (sfGFP T5 target)	

	(https://benchling.com/s/seq-1QTBSMIASWyAX91df2UR?m=slm-wUrfYgMUuhc9OPJxhMe0)	
pGB114	AsCas12a crRNA (sfGFP T6 target) (https://benchling.com/s/seq-F6jtkoAhqO80c2l66Rde?m=slm-MSVI1BDntwCFyDpOReB8)	
pGB115	GoCas12m crRNA (sfGFP T1 target) (https://benchling.com/s/seq-nlUJZzqqD4w5fJPLIHdv?m=slm-Rux4bw37DXPmTGlnhgbQ)	
pGB116	GoCas12m crRNA (sfGFP T2 target) (https://benchling.com/s/seq-JYAzmgc9Jl6FrRRWWafv?m=slm-Sn50ycgHzoUMuCqHtdcq)	
pGB117	GoCas12m crRNA (sfGFP T3 target) (https://benchling.com/s/seq-FhFLANdECkLMwlTEIFhb?m=slm-ObVHiANptVokukbIpex5)	
pGB118	GoCas12m crRNA (sfGFP T4 target) (https://benchling.com/s/seq-bNfrlm1v2RYUQ7Kf0ShJ?m=slm-HNTZleZKVVCg54DRCGVw)	
pGB119	GoCas12m crRNA (sfGFP T5 target) (https://benchling.com/s/seq-fzWdITR2xjop9G12YzBO?m=slm-Qlf1Dfn7OCLZCTYc1WTC)	
pGB120	GoCas12m crRNA (sfGFP T6 target) (https://benchling.com/s/seq-rSPkxQUHHtYgQtuHBSwl?m=slm-HnMTIWJIMfxsdbGrpLW9)	
	Bacteriophage plaque assay in E. coli	
pRZ166	AsCas12a crRNA (non-targeting) (https://benchling.com/s/seq-yaKOvcb1zfp9MuRHwZ82?m=slm-brib6CFbBDQbPBpOsPoo)	2D; S6B
pRZ167	GoCas12m crRNA (non-targeting) (https://benchling.com/s/seq-TBlB3mK3QHnUdpL3FkO6?m=slm-sEuUhkRqS6PkfHy0aP80)	
pGB085	AsCas12a crRNA (T4 phage gp43 sense strand (S) target) (https://benchling.com/s/seq-uvMZNkPdBtd2wgTrRvYS?m=slm-qMW4aGvpvEnKr43qef96)	
pGB086	AsCas12a crRNA (T4 phage gp43 antisense strand (A) target) (https://benchling.com/s/seq-VLBixXI7bQZaHzwjozYr?m=slm-M66ltknwXgMwGfYpf28u)	
pGB087	AsCas12a crRNA (VpaE1 phage gp083 S target) (https://benchling.com/s/seq-5vwgRAw2Hil6kuQXNARc?m=slm-7MqOPgbam4mxTnroSlY1)	
pGB088	AsCas12a crRNA (VpaE1 phage gp083 A target) (https://benchling.com/s/seq-o4UFSx23ztCqRdDxGWvE?m=slm-wo9pY3MyCkosdbpEtud9)	
pGB089	AsCas12a crRNA (λ phage ppB S target) (https://benchling.com/s/seq-77NLpLSCFbd1cfoCV3b0?m=slm-FQDMrR81eAM4kE2WRR7h)	
pGB090	AsCas12a crRNA (λ phage mcpE A target) (https://benchling.com/s/seq-2JbAnKNqpdL1GJCR2IqU?m=slm-jHJHw6RYA4gt44NbVrWg)	
pGB091	AsCas12a crRNA (M13mp18 phage II S target) (https://benchling.com/s/seq-u9GEc1yuJIpXgghWhxaW?m=slm-z7J0LmQY9i2zR1dYLotS)	
pGB092	AsCas12a crRNA (M13mp18 phage II A target) (https://benchling.com/s/seq-XdUdJ3qnO3DBo0cLMG9p?m=slm-FTYb2rJ6OSQiwu2mMGSE)	
pGB093	GoCas12m crRNA (T4 phage gp43 S target) (https://benchling.com/s/seq-9Usv39cfLAsCqboHktFr?m=slm-i1Na7GIEEE20xe63wps7)	

pGB094	GoCas12m crRNA (T4 phage gp43 A target) (https://benchling.com/s/seq-0OD0yuc9SzAMj2xqLJWH?m=slm-IGZgjUQDakxa38y0VUIV)	
pGB095	GoCas12m crRNA (VpaE1 phage gp083 S target) (https://benchling.com/s/seq-1BwFr53T00JQz4kcQQOb?m=slm-g5AIN8kxUL8t6rAk03mX)	
pGB096	GoCas12m crRNA (VpaE1 phage gp083 A target) (https://benchling.com/s/seq-6ldC1KaExuCdpn1jtyOs?m=slm-EsH9ovT1eOBnD7dzTHgA)	
pGB097	GoCas12m crRNA (λ phage ppB S target) (https://benchling.com/s/seq-R021k53uJblepfCSuJDw?m=slm-GWZ2iBt8ZV78OGadLYNe)	
pGB098	GoCas12m crRNA (λ phage mcpE A target) (https://benchling.com/s/seq-r3pECNaM5zw8D0zuD0xH?m=slm-NVvLNfV5mITPUligCL0v)	
pGB099	GoCas12m crRNA (M13mp18 phage II S target) (https://benchling.com/s/seq-mBRSgaoOUslghYJvTQWO?m=slm-Pg2Jgg6H9cAvHwlditcT)	
pGB100	GoCas12m crRNA (M13mp18 phage II A target) (https://benchling.com/s/seq-hcF728ly9CS2wkgS0led?m=slm-VENovvPZprxhMZM8Qlon)	
Base editors expression in E. coli		
pGB129	TadA-8e-GoCas12m (GoABE) and crRNA (CmR target) (https://benchling.com/s/seq-dKV46ZWC9ec3Bp25QOYF?m=slm-a1h5vGOBt3dhtRBVlmO6)	4B; 4C
pGB130	TadA-8e-enAsCas12a (enAsABE) and crRNA (CmR target) (https://benchling.com/s/seq-jCzBRrBCAvI7NnmUuNaq?m=slm-Rvv6vmbOP28IelqBoFF9)	
pGB131	TadA-8e-GoCas12m (GoABE) and crRNA (non-targeting) (https://benchling.com/s/seq-FX68V52ZJssxLcStA45x?m=slm-7KnwggnpEisl0yfsRX2e)	
pGB132	TadA-8e-enAsCas12a (enAsABE) and crRNA (non-targeting) (https://benchling.com/s/seq-ZDLi7gLOnW7Gn8jG0pIF?m=slm-GktSGS4r3cwtyNOVX1zl)	
Chloramphenicol resistance recovery assay in E. coli		
pACYC184	Control target plasmid with intact CmR gene (https://benchling.com/s/seq-VZzlN6qavaUhonOUEVAw?m=slm-yPf2OXIF9dlwcx4rk0np)	4B; 4C
pGB121	Target plasmid with inactive CmR gene (https://benchling.com/s/seq-whv10Q7fTdCy9kj8mHOl?m=slm-Bi23KWl02IfPBpCbrD0R)	
Base editors expression in human cells		
pTK221	TadA-8e-enAsCas12a (enAsABE) (https://www.addgene.org/138506/)	4E; 4F; S11; S12
pTK225	TadA-8e-GoCas12m (GoABE) (https://benchling.com/s/seq-kMTeJ6B2Jj56BCfEh7Pn?m=slm-q1f41MpWbF0viYdxqQ2j)	
eGFP recovery assay in human cells		
pRZ174	Control target plasmid with intact eGFP gene (https://benchling.com/s/seq-KmKR2ZdiTelLkBEnrpvu?m=slm-PNkaSILdmopbRCCF9iAy)	2E; S11
pGB122	Target plasmid with inactive eGFP gene (https://benchling.com/s/seq-T0TdlB33sZ4kpUD5LnUC?m=slm-yok0sLcmehJgCH8duNYs)	

pBD001	GoCas12m crRNA (non-targeting) (https://benchling.com/s/seq-bxtI2SAt0Z9Mbr2GjfUX?m=slm-UL5SOTa410TsJBWO8yxf)	
pBD002	AsCas12a crRNA (non-targeting) (https://benchling.com/s/seq-OYAMATwsKt6WPuaThGih?m=slm-wpMpJ8rHiNb6K2lICH17)	
pGB125	GoCas12m crRNA (eGFP target) (https://benchling.com/s/seq-eB8liHxUlIo1BZ7ij6dJ?m=slm-opQshqn0h1JcuA3YFwSg)	
pGB126	AsCas12a crRNA (eGFP target) (https://benchling.com/s/seq-pWdUVkDzOzGJCdZx7g9i?m=slm-oxDTeBwdpEhNP84joIYY)	
Base editing assay in human cells		
pBD001	GoCas12m crRNA (non-targeting) (https://benchling.com/s/seq-bxtI2SAt0Z9Mbr2GjfUX?m=slm-UL5SOTa410TsJBWO8yxf)	2F; S12
pBD002	AsCas12a crRNA (non-targeting) (https://benchling.com/s/seq-OYAMATwsKt6WPuaThGih?m=slm-wpMpJ8rHiNb6K2lICH17)	
pBD005	GoCas12m crRNA (16 target) (https://benchling.com/s/seq-Zw8zIvzX5M6xvWn1N3GT?m=slm-XSP7cBh3Gy5RKPQyIoWt)	
pBD006	GoCas12m crRNA (18 target) (https://benchling.com/s/seq-Y2gNwbAuhINObSinMQQF?m=slm-Sa3IgoM7IdqL1naR0dcl)	
pBD009	AsCas12a crRNA (16 target) (https://benchling.com/s/seq-a4p3KQJWR50ZT6z3VdVn?m=slm-0Zh2cLw6O0nnQafTyu67)	
pBD010	AsCas12a crRNA (18 target) (https://benchling.com/s/seq-joFnsT0370QHSy3dSimp?m=slm-M6cQdQxslif0OsksjNpT)	
pBD017	GoCas12m crRNA (17 target) (https://benchling.com/s/seq-x43CkI1bb1WQwuwa8frU?m=slm-OXwStJTzDxUz2abhhbBM)	
pBD018	AsCas12a crRNA (17 target) (https://benchling.com/s/seq-NZN53QEeOkAHSHRTAN3D?m=slm-82pTTTsA3nwpEyeQc3Ey)	
pBD023	GoCas12m crRNA (13 target) (https://benchling.com/s/seq-9bcGH45Kaq9tEWxE77IX?m=slm-CsTngrtsJQEpY64zWFMZ)	
pBD024	AsCas12a crRNA (13 target) (https://benchling.com/s/seq-0UdixwlFig08Wq1Wla0O?m=slm-N3RGdX9FPmmVNWyxQ2QQ)	
pBD025	GoCas12m crRNA (14 target) (https://benchling.com/s/seq-H7SWeVu9DvsrOXdQD5jJ?m=slm-sue8O0NrrrqEapEcTfYH)	
pBD026	AsCas12a crRNA (14 target) (https://benchling.com/s/seq-hv0GDUcAklzShYZLCnP8?m=slm-6Wiy1S0RBVurCBek6rtL)	

Table S5. RNAs used in this study.

	pre-crRNA	
Cas12m	Sequence 5’-3’ (repeat, spacer)	Figures
Ms	GGGCUGGACGUAUUGUAGGAUGGGGGGAGUAGCAACGCCUAGCUCAGGGGCUCGAAAACUGAGACAAGUUGACCCA ACGUCGCCGGCGUGCACAAUCUAGA	S2B
	crRNA	
Cas12m	Sequence 5’-3’ (repeat, spacer)	Figures
Ms	GGGUAGCAACGCCUAGCUCAGGGGCUCGAAAACUGAGACAAGUUGACCCAACGUCGCCGG	1D; S3; S5
Tf	GGGUUGCAAUGCCUAGCUCAGAGGUUUAAAGACUGAGACAGUUGACCCAACGUCGCCGG	1D; S3
Ap	GGGUGACAAAGCCCUGUGCAGCGGGCUCAAAGCUGCGACAGUUGACCCAACGUCGCCGG	1D; S3
Un	GGGUUGCAAUGCCUGGCUCAGGGGUAUAAGAACUGAGACAGUUGACCCAACGUCGCCGG	1D; S3
Go	GGGUGUCAACGCCAGCGCGGAGGCGUCAAAUCCGCGACAGUUGACCCAACGUCGCCGG	1D; 3; S3; S4A S5; S7; S8; S9
	GGGUGUCAACGCCAGCGCGGAGGCGUCAAAUCCGCGACCGUGCGUGGCGAGGGUGAAG	S4C
Mm	GGGUGUCAUAGCCCAGCUUGGCGGGCGAAGGCCAAGACAGUUGACCCAACGUCGCCGG	1D; S3
Pp	GGGUUGACAUGCGUGCUCCGCCGCUUUGUAGUGGAGACAGUUGACCCAACGUCGCCGG	1D; S3
Mk	GGGUUACAAACCCUGCUCAUUGGGUUGGUUAAUGAGACAGUUGACCCAACGUCGCCGG	1D; S3
Cas12a	Sequence 5’-3’ (repeat, spacer)	Figures
As	GGGUAAUUUCUACUCUUGUAGAUAGUUGACCCAACGUCGCCGGCGU	S5
	RNA targets	
Target name	Sequence 5’-3’ (target)	Figures
T	GGGCAGCUGAUGCAUCUAGAUUGUGCACGCCGGCGACGUUGGGUCAACUUAAAUACGCUACCUUGCAGUAAGGUGC	S5B
NT	GGGCAGCUGAUGCAUCUAGAUUGUGCACGUCAUGGGCGCCAAGGACUCAUCAAGCGCUACCUUGCAGUAAGGUGC	
	RNA marker	
Length	Sequence 5’-3’	Figures
101 nt	GGGCUGGACGUAUUGUAGGAUGGGGGGAGUAGCAACGCCUAGCUCAGGGGCUCGAAAACUGAGACAAGUUGACCCA ACGUCGCCGGCGUGCACAAUCUAGA	S2B
76 nt	GGGCAGCUGAUGCAUCUAGAUUGUGCACGCCGGCGACGUUGGGUCAACUUAAAUACGCUACCUUGCAGUAAGGUGC	
68 nt	AGCGGUGGAAAACAACCACCUUAUUCACUUGUCUUAAUUUGUCAAUGCUUUAGGAGUUAAACGGCGGG	
42 nt	CGCUAAAGAGGAAGAGGACAGUUUUAGAGCUGUGUUGUUUCG	
31 nt	GGGCAAGGAGGUAAAAAUGUAGAAAAACAAU	
16 nt	ACGGAAACUUUCGUAA	

Table S6. DNA oligonucleotides used in this study.

Description	Sequence 5'-3' (PAM, target)	Figures
	AsCas12a, dAsCas12a, GoCas12m	
Target (forward)	GCACCTTACTGCAAGGTAGCGTATTTAAGTTGACCCAACGTCGCCGGCGTGCACAATCTAGATGCATCAGCTGC	S5A
Target (reverse)	GCAGCTGATGCATCTAGATTGTGCACGCCGGCGACGTTGGGTCAACTTAAATACGCTACCTTGCAGTAAGGTGC	
ssDNA activator	GCAGCTGATGCATCTAGATTGTGCACGCCGGCGACGTTGGGTCAACTTAAATACGCTACCTTGCAGTAAGGTGC	S5C; S5D
dsDNA activator	GCACCTTACTGCAAGGTAGCGTATTTAAGTTGACCCAACGTCGCCGGCGTGCACAATCTAGATGCATCAGCTGC	
	MsCas12m	
Target (forward)	GCACCTTACTGCAAGGTAGCGTATTTTAGTTGACCCAACGTCGCCGGCGTGCACAATCTAGATGCATCAGCTGC	S5A
Target (reverse)	GCAGCTGATGCATCTAGATTGTGCACGCCGGCGACGTTGGGTCAACTAAAATACGCTACCTTGCAGTAAGGTGC	
ssDNA activator	GCAGCTGATGCATCTAGATTGTGCACGCCGGCGACGTTGGGTCAACTAAAATACGCTACCTTGCAGTAAGGTGC	S5C; S5D
dsDNA activator	GCACCTTACTGCAAGGTAGCGTATTTTAGTTGACCCAACGTCGCCGGCGTGCACAATCTAGATGCATCAGCTGC	
Forward strand marker	GCACCTTACTGCAAGGTAGCGTATTTAAGTTGACCCAACGTCGCCGGCGTGCACAATCTA	S5A
	GCACCTTACTGCAAGGTAGCGTATTTAAGTTGACCCAACGTCGCCGGCGT	
	GCACCTTACTGCAAGGTAGCGTATTTAAGTTGACCCAACG	
	GCACCTTACTGCAAGGTAGCGTATTTAAGT	
	GCACCTTACTGCAAGGTAGC	
	GCACCTTACT	
Reverse strand marker	GCAGCTGATGCATCTAGATTGTGCACGCCGGCGACGTTGGGTCAACTTAAATACGCTACC	S5A
	GCAGCTGATGCATCTAGATTGTGCACGCCGGCGACGTTGGGTCAACTTAA	
	GCAGCTGATGCATCTAGATTGTGCACGCCGGCGACGTTGG	
	GCAGCTGATGCATCTAGATTGTGCACGCCG	
	GCAGCTGATGCATCTAGATT	
	GCAGCTGATG	
	Adapter used for dsDNA cleavage activity testing	
Forward	GATCGGAAGAGCGGTTCAGCAGGAATGCCG	S3
Reverse	CGGCATTCCTGCTGAACCGCTCTTCCGATCT	
	DNA used for BLI experiments	
Target (forward)	b-GCACCTTACTGCAAGGTAGCGTATTTAAGTTGACCCAACGTCGCCGGCGTGCACAATCTAGATGCATCAGCTGC	S4A
Target (reverse)	GCAGCTGATGCATCTAGATTGTGCACGCCGGCGACGTTGGGTCAACTTAAATACGCTACCTTGCAGTAAGGTGC	
Target (forward)	b-GCACCTTACTGCAAGGTAGCGTAGCCTAGTTGACCCAACGTCGCCGGCGTGCACAATCTAGATGCATCAGCTGC	
Target (reverse)	GCAGCTGATGCATCTAGATTGTGCACGCCGGCGACGTTGGGTCAACTAGGCTACGCTACCTTGCAGTAAGGTGC	
Target (forward)	b-GCACCTTACTGCAAGGTAGCGCTTGATGAGTCCCTTGGCGCCCATGACGTGCACAATCTAGATGCATCAGCTGC	
Target (reverse)	GCAGCTGATGCATCTAGATTGTGCACGTCATGGGCGCCAAGGGACTCATCAAGCGCTACCTTGCAGTAAGGTGC	
	DNA used to obtain GoCas12m-crRNA-DNA ternary complex for cryo-EM	
1st target strand	CCGGCGACGTTGGGTCAACTGAAACAGACATTTCTCAACAAAAA	3; S7; S8; S9
2nd target strand	CCGGCGACGTTGGGTCAACTGAAATGTCTGTTTCTCAACAAAAA	

Table S7. Primers used in this study.

DNA cleavage-based PAM identification assay		
Name	Sequence 5’-3’	Figures
TK-735	ACACTCTTTCCCTACACGACGCTCTTCCGATCTCGGCATTCCTGCTGAAC	S3
TK-737	GTGACTGGAGTTCAGACGTGTGCTCTTCCGATCTACTATAGGGAAAGCTTGCATG	
TK-765	ACACTCTTTCCCTACACGACGCTCTTCCGATCTGCGGCATTCCTGCTGAAC	
TK-766	ACACTCTTTCCCTACACGACGCTCTTCCGATCTTGCGGCATTCCTGCTGAAC	
TK-767	ACACTCTTTCCCTACACGACGCTCTTCCGATCTATGCGGCATTCCTGCTGAAC	
TK-768	ACACTCTTTCCCTACACGACGCTCTTCCGATCTCATGCGGCATTCCTGCTGAAC	
TK-769	ACACTCTTTCCCTACACGACGCTCTTCCGATCTGCATGCGGCATTCCTGCTGAAC	
TK-770	ACACTCTTTCCCTACACGACGCTCTTCCGATCTTGCATGCGGCATTCCTGCTGAAC	
TK-771	ACACTCTTTCCCTACACGACGCTCTTCCGATCTATGCATGCGGCATTCCTGCTGAAC	

DNA binding-based PAM identification assay		
Name	Sequence 5’-3’	Figures
TK-736	ACACTCTTTCCCTACACGACGCTCTTCCGATCTTCGGTACCTCGCGAATG	1D
TK-737	GTGACTGGAGTTCAGACGTGTGCTCTTCCGATCTACTATAGGGAAAGCTTGCATG	
TK-772	ACACTCTTTCCCTACACGACGCTCTTCCGATCTGTCGGTACCTCGCGAATG	
TK-773	ACACTCTTTCCCTACACGACGCTCTTCCGATCTTGTCGGTACCTCGCGAATG	
TK-774	ACACTCTTTCCCTACACGACGCTCTTCCGATCTATGTCGGTACCTCGCGAATG	
TK-775	ACACTCTTTCCCTACACGACGCTCTTCCGATCTCATGTCGGTACCTCGCGAATG	
TK-776	ACACTCTTTCCCTACACGACGCTCTTCCGATCTGCATGTCGGTACCTCGCGAATG	
TK-777	ACACTCTTTCCCTACACGACGCTCTTCCGATCTTGCATGTCGGTACCTCGCGAATG	
TK-778	ACACTCTTTCCCTACACGACGCTCTTCCGATCTATGCATGTCGGTACCTCGCGAATG	

Magnetic tweezers experiments		
JMM_For_NotI	CCTAAAGGCTGCGGCCGCACCCTCGCAAGC	S5
JMM_Rev_HindIII	CAACTGGCTGACAAGCTTTATGCCTCTCC	

Base editing assay			
Target site	Name	Sequence 5’-3’	Figures
13	GB-1157	CCTACACGACGCTCTTCCGATCTCCCAGGGAAACGCCCATGC	4F; S12
	GB-1158	TTCCTTGGCACCCGAGAATTCCAAAGGATTGACCCAGGCCAGG	
14	GB-1159	CCTACACGACGCTCTTCCGATCTTGGCAGAGGAAAGGAAGCCC	
	GB-1160	TTCCTTGGCACCCGAGAATTCCAACTTGTCAACCAGTATCCCG	
16	GB-1000	CCTACACGACGCTCTTCCGATCTGGTGACCCATTTCCATTCAAGG	
	GB-1001	TTCCTTGGCACCCGAGAATTCCAGTGAGGAGAAGGCAGGAGG	
17	GB-1151	CCTACACGACGCTCTTCCGATCTATAACGAAGAACTCTTTGTGG	
	GB-1152	TTCCTTGGCACCCGAGAATTCCAAAAGGACATACGGGGAGG	
18	GB-1004	CCTACACGACGCTCTTCCGATCTTTTATTGTTTTGTTTTCCTCCTGG	

	GB-1005	TTCCTTGGCACCCGAGAATTCCAGCTTTTTTGTGCGTGCTTC	

Table S8. Target sequences used in this study.

Plasmid interference assay in E. coli			
Cas12	Target name	Sequence 5'-3' (PAM, target)	Figures
AsCas12a	-	-GGGTCTTCGAGAAGACAC	2A; S6A
	T2	TTTAAGTTGACCCAACGTCGCCGGCGT	
	KanR (T3)	TTTATATGGGTATAAATGGGCTCGCGA	
	Ori (T1)	TTTCCTTTGAGTTGTGGGTATCTGTAA	
GoCas12m	-	-GGGTCTTCGAGAAGACAC	
	T2	TTTAAGTTGACCCAACGTCGCCGGCGT	
	KanR (T3)	TTTATATGGGTATAAATGGGCTCGCGA	
	Ori (T1)	TTTCCTTTGAGTTGTGGGTATCTGTAA	
MsCas12m	-	-GGGTCTTCGAGAAGACAC	
	T2	TTAGTTGACCCAACGTCGCCGG	
	KanR (T3)	TTATATGGGTATAAATGGGCTC	
	Ori (T1)	TTCCTTTGAGTTGTGGGTATCT	
Pp&TfCas12m	-	-GGGTCTTCGAGAAGACAC	
	T2	CCTAGTTGACCCAACGTCGCCGG	
	KanR (T3)	CCAACATGGACGCTGATTTATAT	
	Ori (T1)	CCTTTGAGTTGTGGGTATCTGTA	
sfGFP silencing assay in E. coli			
Cas12	Target name	Sequence 5'-3' (PAM, target)	Figures
AsCas12a	-	-GGGTCTTCGAGAAGACAC	2C
	sfGFP T1	TTTCTTGACAGCTAGCTCAGTCCTAGG	
	sfGFP T2	TTTCTCTAGTAGCTAGCACAATACCTA	
	sfGFP T3	TTTCCGTGCGTGGCGAGGGTGAAGGTG	
	sfGFP T4	TTTACCATTAGTTGCGTCACCTTCACC	
	sfGFP T5	TTTAAAATTCGCCACAACGTGGAGGAT	
	sfGFP T6	TTTCTCGTTCGGATCTTTAGACAGAAC	
GoCas12m	-	-GGGTCTTCGAGAAGACAC	
	sfGFP T1	TTTCTTGACAGCTAGCTCAGTCCTAGG	
	sfGFP T2	TTTCTCTAGTAGCTAGCACAATACCTA	
	sfGFP T3	TTTCCGTGCGTGGCGAGGGTGAAGGTG	
	sfGFP T4	TTTACCATTAGTTGCGTCACCTTCACC	
	sfGFP T5	TTTAAAATTCGCCACAACGTGGAGGAT	
	sfGFP T6	TTTCTCGTTCGGATCTTTAGACAGAAC	
Bacteriophage plaque assay			
Cas12	Target name	Sequence 5'-3' (PAM, target)	Figures
AsCas12a	-	-GGGTCTTCGAGAAGACAC	2D; S6B
	T4 S	TTTATATCTCTATTGAAACAGTCGGAA	
	T4 A	TTTGACTCTTCCTTACAATGCCTAAAC	
	VpaE1 S	TTTAGAGGGAGTAAAAATGGAAAACGT	
	VpaE1 A	TTTACTCCCTCTAAAATCATTAACCTC	
	λ S	TTTGGAGGGCAGTTGCGGTCGTGGAAC	
	λ A	TTTGATCCGCTGTTTCTGCGTCTCTTT	
	M13mp18 S	TTTACGATTACCGTTCATCGATTCTCT	
	M13mp18 A	TTTGAGAGATCTACAAAGGCTATCAGG	
GoCas12m	-	-GGGTCTTCGAGAAGACAC	
	T4 S	TTTATATCTCTATTGAAACAGTCGGAA	
	T4 A	TTTGACTCTTCCTTACAATGCCTAAAC	
	VpaE1 S	TTTAGAGGGAGTAAAAATGGAAAACGT	
	VpaE1 A	TTTACTCCCTCTAAAATCATTAACCTC	
	λ S	TTTGGAGGGCAGTTGCGGTCGTGGAAC	
	λ A	TTTGATCCGCTGTTTCTGCGTCTCTTT	
	M13mp18 S	TTTACGATTACCGTTCATCGATTCTCT	
	M13mp18 A	TTTGAGAGATCTACAAAGGCTATCAGG	
Chloramphenicol resistance recovery assay in E. coli			

Cas12-base editor	Target name	Sequence 5'-3' (PAM, target)	Figures
TadA-8e-enAsCas12a	-	-GGGTCTTCGAGAAGACAC	4B; 4C
	CmR	TTTCCGATGCCATTAGGATATATCAAC	
TadA-8e-GoCas12m	-	-GGGTCTTCGAGAAGACAC	
	CmR	TTTCCGATGCCATTAGGATATATCAAC	
GFP recovery assay in human cells			
Cas12-base editor	Target name	Sequence 5'-3' (PAM, target)	Figures
TadA-8e-enAsCas12a	-	-AGAAGAGCTCTAGCTCTTCC	4E; S11
	ΔeGFP	TTTCACCTCGGCTCAGGTCTTGTAGTT	
TadA-8e-GoCas12m	-	-AGAAGAGCTCTAGCTCTTCC	
	ΔeGFP	TTTCACCTCGGCTCAGGTCTTGTAGTT	
Base editing assay in human cells			
Cas12-base editor	Target site	Sequence 5'-3' (PAM, target)	Figures
TadA-8e-enAsCas12a	-	-AGAAGAGCTCTAGCTCTTCC	4F; S12
	13	TTTCTGCTGCAAGTAAGCATGCATTTG	
	14	TTTCCTAGACAGGGGCTAGTATGTGCA	
	16	TTTAGAAGCACATCAAGGACATTCTAA	
	17	TTTGGGATAAGCACAGTTTTAAATAGT	
	18	TTTGGTTTAAACACACCGGGTTAATAA	
TadA-8e-GoCas12m	-	-AGAAGAGCTCTAGCTCTTCC	
	13	TTTCTGCTGCAAGTAAGCATGCATTTG	
	14	TTTCCTAGACAGGGGCTAGTATGTGCA	
	16	TTTAGAAGCACATCAAGGACATTCTAA	
	17	TTTGGGATAAGCACAGTTTTAAATAGT	
	18	TTTGGTTTAAACACACCGGGTTAATAA	

Table S9. Cryo-EM data collection, refinement and validation statistics for GoCas12m-crRNA-DNA ternary complex.

GoCas12m-crRNA-DNA ternary complex (EMDB-17757) (PDB 8PM4)	
**Data collection and processing**	
Magnification	92,000
Voltage (kV)	200
Electron exposure (e-/Å^2)	29.7
Tilt angle	0
Defocus range (μm)	−1.0 to −2.0
Pixel size (Å)	1.1
Symmetry imposed	C1
Initial particles images (no.)	1,559,728
Final particles images (no.)	204,822
Sphericity	0.94
Map resolution (Å)	2.93
FSC threshold	0.143
Map resolution range (Å)	2.47-35.19
**Refinement**	
Initial model used	AlphaFold
Model resolution ($d_{FSC\ model}$) (Å)	3.0
FSC threshold	0.143
Map sharpening *B*-factor (Å^2)	-127.34
Model composition	
Non-hydrogen atoms	6624
Protein residues	604
Nucleotides	96
Ligands	0
Water	0
B-factors (Å^2) min/max/mean	
Protein	20.58/160.45/68.22
Nucleotides	9.09/152.06/63.47
R.m.s. deviations	
Bond lengths (Å)	0.007
Bond angels (°)	0.634
**Validation**	
MolProbity score	1.65
Clashscore	11.13
Poor rotamers (%)	1.32
CaBLAM outliers (%)	0.5
Cβ outliers (%)	0
Peptide plane (%)	
Cis proline/general	3.6/0.0
Twisted proline/general	0.0/0.0
Ramachandran plot	
Favored (%)	98.34
Allowed (%)	1.66
Disallowed (%)	0

Table S10. Flow cytometry results.

			GFP positive, %		
Base editor	crRNA	Replicate	24 h	48 h	72 h
-	-	1	0.43	0.46	0.48
enAsABE	Non-targeting	1	0.26	2.37	2.75
		2	0.28	2.30	2.33
		3	0.25	2.26	2.19
	Targeting	1	17.80	51.00	33.80
		2	17.70	46.10	38.70
		3	16.40	42.40	35.60
GoABE	Non-targeting	1	0.36	3.71	2.71
		2	0.51	3.60	3.24
		3	0.40	5.10	3.73
	Targeting	1	12.70	40.90	31.40
		2	12.90	40.40	34.00
		3	10.90	38.80	32.50

REFERENCES

1. Sasnauskas,G., Tamulaitiene,G., Druteika,G., Carabias,A., Silanskas,A., Kazlauskas,D., Venclovas,Č., Montoya,G., Karvelis,T. and Siksnys,V. (2023) TnpB structure reveals minimal functional core of Cas12 nuclease family. *Nature*, **616**, 384–389.

2. Richter,M.F., Zhao,K.T., Eton,E., Lapinaite,A., Newby,G.A., Thuronyi,B.W., Wilson,C., Koblan,L.W., Zeng,J., Bauer,D.E., *et al.* (2020) Phage-assisted evolution of an adenine base editor with improved Cas domain compatibility and activity. *Nat. Biotechnol.*, **38**, 883–891.
